# ATMO-vent: An adapted breathing atmosphere for COVID-19 patients

**DOI:** 10.1016/j.ohx.2020.e00145

**Published:** 2020-09-26

**Authors:** Thasshwin Mathanlal, Miracle Israel Nazarious, Roberto Mantas-Nakhai, Maria-Paz Zorzano, Javier Martin-Torres

**Affiliations:** a Group of Atmospheric Science, Department of Computer Science, Electrical and Space Engineering, Luleå, University of Technology, Luleå 97 187, Sweden; b Centro de Astrobiología (CSIC-INTA), Torrejon de Ardoz, 28850 Madrid, Spain; c Instituto Andaluz de Ciencias de la Tierra (CSIC-UGR), 18100 Granada, Spain; d School of Geosciences, University of Aberdeen, Meston Building, King's College, Aberdeen AB24 3UE, UK

**Keywords:** Mechanical ventilator, Air-oxygen mixing, Low-cost, Rapid development time, Commercial off the shelf

## Abstract

The ongoing worldwide pandemic of coronavirus disease 2019 (COVID-19), has been one of the most significant challenges to humankind in centuries. The extremely contagious nature of the SARS-CoV-2 virus has put forth an immense pressure on the health sector. In order to mitigate the stress on the healthcare systems especially to battle the crisis of mechanical ventilators, we have designed a modular, and robust DIY ventilator, ATMO-Vent (Atmospheric Mixture Optimization Ventilator) which can be fully mounted within two days by two operators. The ATMO-Vent has been designed using low-cost, robust, Commercial Off The Shelf (COTS) components, with many features comparable to a full-fledged ventilator. ATMO-Vent has been designed based on the United Kingdom Medicines & Healthcare products Regulatory Agency (UK-MHRA) guidelines for Rapidly Manufactured Ventilator System (RMVS), yet is scalable to the specific requirements of different countries. ATMO-Vent is capable of adjusting the Fraction of Inspiratory Oxygen (FiO_2_) levels, Tidal Volume (TV), frequency of breaths, Inspiratory/Expiratory ratio (I/E), Peak Inspiratory Pressure (PIP) and Positive End-Expiratory Pressure (PEEP). ATMO-Vent can operate in two modes – Continuous Mandatory Ventilation (CMV) using Volume-Controlled Ventilation (VCV) and in Assisted Control (AC) mode with pressure triggered by the patient. ATMO-Vent has undergone rigorous testing and qualifies under Class B Electric and Magnetic Compatibility (EMC) requirements of EN 55,011 CISPR 11 standards.

## Graphical abstract

**Figure:**
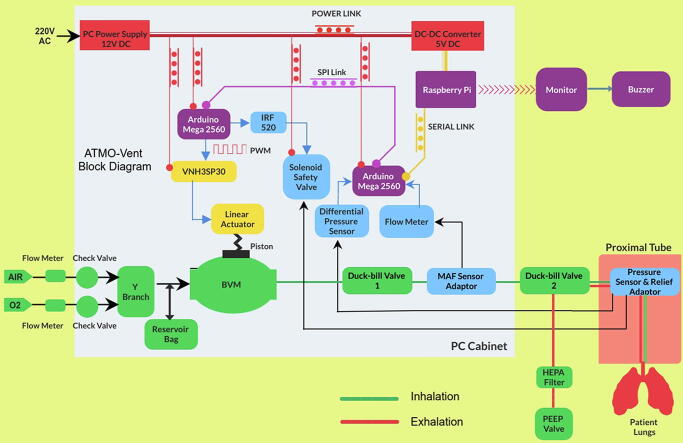

## 1 Specifications table

**Table:** 

Hardware name	ATMO-Vent (Atmospheric Mixture Optimization Ventilator)
Subject area	• Medical
Hardware type	• Life-saving equipment
Open Source License	GNU General Public License (GPL) 3.0
Cost of Hardware	1000 GBP
Source File Repository	https://data.mendeley.com/datasets/2c4cwmvcs8/1

## 2 Hardware in context

Mechanical ventilators are crucial for patients who develop Acute Respiratory Distress Syndrome (ARDS), a sudden and severe lung failure, and with the current pandemic situation, the need for them is increasing day by day with tens of thousands of people being infected by SARS-CoV-2. A mechanical ventilator pushes air, using an endotracheal tube (ETT), in and out of a patient’s lungs. The ventilator itself does not cure the patient of the COVID-19 disease, but they are useful in the patient’s recovery along with other antibiotics treatments (in case of microbial infection) and patient’s immunological system. Many research groups, companies, and individuals have already responded to the call from different governments to the need for ventilators by creating new innovative designs. For example, the companies in the VentilatorChallengeUK consortium have received formal orders from the UK Government for more than 15,000 ventilator units. When applied, mechanical ventilators may be typically needed for a couple of weeks, and this requires about a million cycles of assisted ventilation. Mechanical ventilators should be used under the supervision of a qualified licensed health professional. During this time, the configuration of the ventilator needs to be adapted, for diagnosis and evaluation and to be readapted to the evolving condition of the patient, which may eventually lead to retire the supporting ventilation. Therefore, not only the robustness and industrial compliance of any mechanical ventilator needs to be certified according to the local industrial and medical standards, but also the information displayed in the screen, and the operating software interface has to be adapted so that the professionals that are manipulating it can rapidly and intuitively implement their tasks and also that alarms can warn the user when specific values get out of their nominal range. The purpose of this article is to share in an open format, the mechanical and electrical instructions and the software required to build and operate ATMO-Vent (Atmospheric Mixture Optimization Ventilator), a mechanical ventilator that can be entirely made with Commercial Off The Shelf (COTS) components. This will serve to provide bounds of the cost and the time of development per unit for future applications. This document also provides specific components, assembly, and operating instructions for any institution who may need to construct such a device.

ATMO-Vent can be used as both a non-invasive positive pressure ventilator (NPPV) with facial masks or as an invasive ventilator with intubation using an endotracheal tube. ATMO-Vent has been designed with adherence to the guidelines elucidated in the United Kingdom Medicines & Healthcare products Regulatory Agency (UK-MHRA) guidelines for Rapidly Manufactured Ventilator System (RMVS) [1]. Few healthcare regulation agencies such as Spanish Agency of Medicines and Medical Devices (AEMPS) of Spain and United States Food and Drug Administration (US FDA) have also issued guidelines for developing such emergency use ventilators, but with the absence of a global standard for design of open source ventilators, the UK RMVS guidelines are used as the baseline standard for development of such ventilators. Safety and easy operability by healthcare professionals are the two main factors considered in ATMO-Vent development. In terms of safety, most of the components of the ventilator mainly used in-line with the patient’s airway are approved by the Food and Drug Administration (FDA) and are medically compatible. The ventilator interface has been designed in such a way that it requires minimal training to operate the ventilator. ATMO-Vent mimics some of the modes found in a full-fledged mechanical ventilator like the volume-control Continuous Mandatory Ventilation (VC-CMV) and Assisted Control (VC-AC) modes in Hamilton C1 and Dräger Savina 300 ventilators. The ATMO-Vent DIY design is unique compared to the similar open-source DIY designs currently available, in its ability to control various respiration parameters and the modes of operation. The design utilizes the Bag Valve Mask (BVM) as the core respiration component similar to the designs of E-Vent of MIT [2], OxVent of University of Oxford and King’s College London [3] and ApolloBVM from Rice University [4]. The ATMO-Vent uses a linear actuator to actuate the BVM, which provides higher versatility in controlling the respiration parameters such as the Tidal Volume (TV), frequency of breaths, Peak Inspiratory Pressure (PIP) and Positive End-Expiratory Pressure (PEEP). The Graphical User Interface (GUI) of ATMO-Vent is very similar to that of the existing, commercial, full-fledged mechanical ventilator with the ability to display respiratory parameters such as Plateau Pressure, Mean Airway Pressure, Minute Ventilation, Resistance and Compliance which are crucial for healthcare professionals to determine the lung condition of the patient. Adhering to the RMVS guidelines, ATMO-Vent features a CMV Mode which minimally is a VCV in addition to an AC-VCV which can be pressure triggered by the patient. The former mode is used as invasive ventilation with intubation for people with ARDS, and the latter can be used as non-invasive positive pressure ventilation for people with mild to moderate respiratory discomfort. ATMO-Vent incorporates safety features such as audio-visual alarms when the frequency of breaths is below the set threshold, pressure in airway exceeds the PIP or if the set TV is not delivered to the patient. In case of airway pressure exceeding the set PIP, the linear actuator movement immediately ceases, and the solenoid actuating valves opens to vent any excess pressure. All these features are designed using commercial open-source electronics platforms such as Arduino and Raspberry Pi along with medical device compatible sensors. A robust construction along with a carefully designed software architecture in compliance with the RMVS guidelines adhering to modes of ventilation, infection control, safety alarms and biological contamination make ATMO-Vent a unique DIY solution to battle the current ventilator crisis with a minimal investment of time, financial and human resources.

## 3 Hardware description

**Fig. 1:**
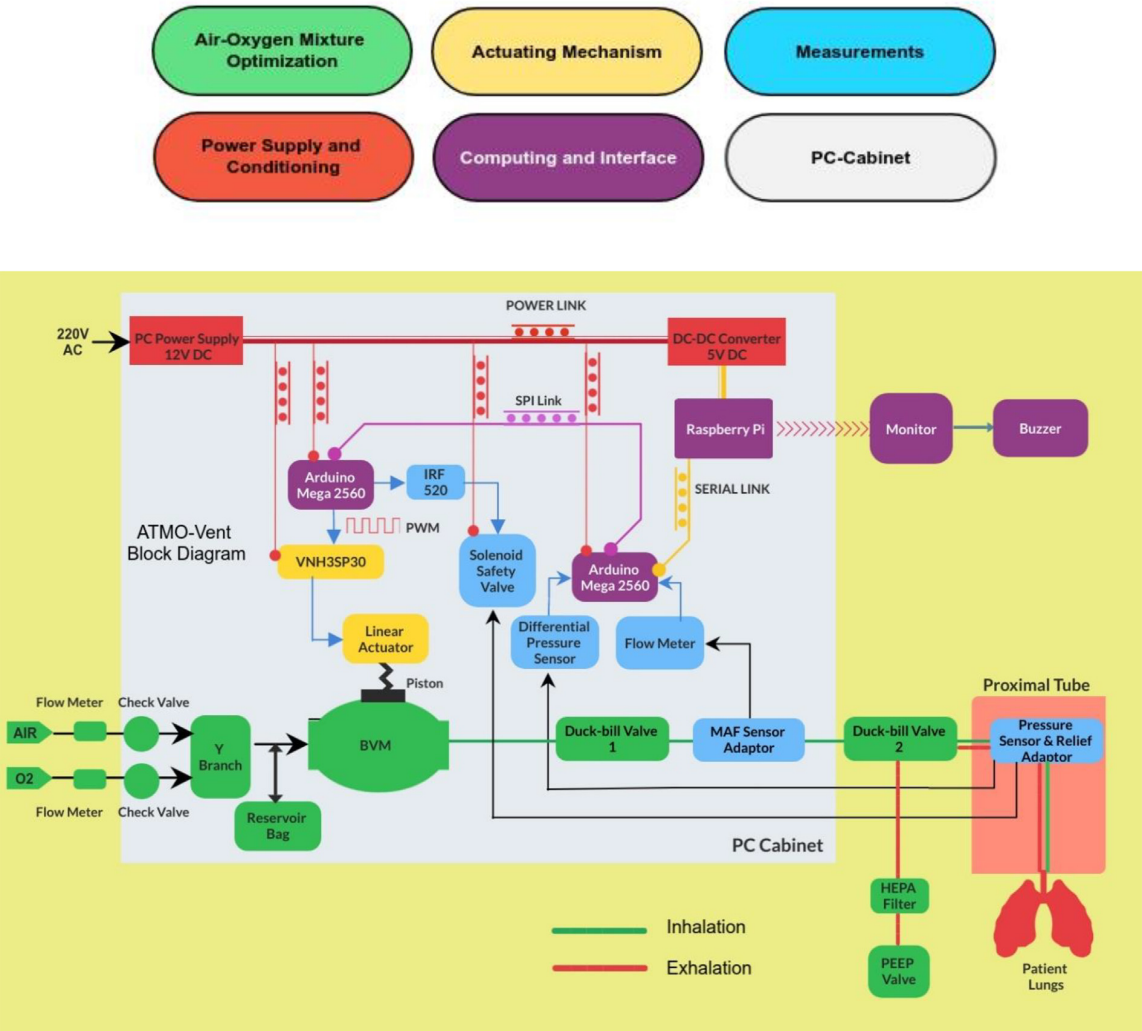
ATMO-Vent block diagram with six colour-coded sub-modules.

ATMO-Vent is a BVM based ventilator which has been completely designed using readily available COTS components. The materials used have been meticulously chosen such that there is a very minimal adaptive work needed to fit the ventilator application. The components used in-line with the patient’s airway, has been constrained purely to medically approved and compatible elements. ATMO-Vent uses an efficient design construction utilizing minimal components without compromising the safety and operability of the ventilator. Based on the Harvard EdX course on mechanical ventilation [5], the modes of operation programmed in ATMO-Vent were implemented with an efficient GUI, resembling the interface of the commercially available Hamilton C1 ventilator. This was done in keeping mind of the ease of training to the clinicians, respiratory therapists and intensive care nurses to operate ATMO-Vent. Fig. 1 shows the architecture block diagram of ATMO-Vent. The block diagram is colour coded in six categories as follows:

### 3.1 Air-oxygen mixture optimization circuit

The Air-Oxygen Mixture Optimization circuit controls the Fraction of Inspiratory Oxygen (FiO_2_). Compressed medical-grade air and oxygen are supplied into the circuit using the 8 mm tubing. The polyurethane tube is FDA approved for use in the food industry. The circuit does not include the humidifier and the pressure regulating valve, which are commonly found in healthcare infrastructure. The flow rates of air and oxygen are controlled using the flow meters. The MR3000 flow meter from Brooks Instruments can control the flow of each gas from 2 LPM to 30 LPM with a full-scale accuracy of ± 4%. The flow meter can handle 6.9 bar pressure of the gas, and it is crucial to ensure that the pressure setting on the pressure regulating valve at the source is below 6.9 bar. The output from the flowmeters is connected to the non-return check valves. The valve prevents the flow of the gas with a higher flow rate into the other path in the reverse direction. The output of the non-return check valves is connected to the Y-branch, where the mixing of air and oxygen takes place. The output of the Y-branch is connected to the oxygen inlet of the BVM. A reservoir is attached to the BVM, to store the excess gas mixture which acts a reservoir during subsequent breaths. The reservoir also has a valve to vent out excess atmosphere to avoid building up of pressure. This ensures a uniform flow of air-oxygen mixture to the patient. The ratio of air and oxygen is controlled by the health care professional based on the patient’s requirements. The BVM used in the ATMO-Vent is designed to accommodate a maximum of 30 LPM air and oxygen flow combined. Over 30 LPM the duck-bill valve would partially open irrespective of being actuated and would increase the resistance against which the patient has to exhale. This limits the current design of ATMO-Vent to deliver a maximum of 30 LPM of pure air or 100% oxygen or any mixture within the 30LPM range without increasing exhalation resistance. We plan to increase the individual flowmeter range to 60LPM each and include a normally closed solenoid valve in the proximal tube after the BVM, such that it only opens when the linear actuator compresses the BVM. This would allow for the average adult ventilation flow rate of 50-60LPM without increasing the exhalation resistance.

Dead space is a critical issue which can limit the gas exchange in the lungs by accumulating carbon dioxide from the patient’s exhaling breath in the long proximal tube between the BVM and the patient. In order to mitigate the dead space, ATMO-Vent uses a double duck-bill valve assembly (one at the output of the BVM and the other at the exhaust as close as possible to the patient) to reduce the proximal tube length. The exhaust of the duck-bill valve assembly close to the patient is fixed with a spring-loaded PEEP valve that maintains an end positive pressure in the airway after exhalation. This positive pressure prevents the alveoli of the lungs from collapsing after a breath. The PEEP valve is connected to the exhaust port using a PEEP adapter through a High-Efficiency Particulate Air (HEPA) filter. The filter ensures that any virus or microbe from the patient’s airway does not spread out into the ambient atmosphere. The HEPA filter is crucial to prevent health care professionals from being infected by the patient. The second duck-bill valve within 15–20 cm from the patient’s face, is the patient inflating valve which allows the flow of air and oxygen mixture from the BVM to ventilate the patient and at the same time closes the expiratory limb. During expiration, the valve closes the inspiratory limb and exhausts the exhaled air through the PEEP valve and HEPA filter assembly. Having the duck-bill valve close to the patient reduces the dead space, which prevents patients from re-breathing excess carbon dioxide, should the duck-bill valve be located far from the patient by increasing the proximal tube length. The Laerdal (duck-bill) and Ambu based patient valves are widely used as non-return valves in Bag Valve Mask (BVM) based resuscitators owing to their simplicity and reliability [6]. The validation of the duck-bill valve as a patient inflating valve has been discussed in the Validation and Characterization section of the article. Duckbill valves in Resuscitation bags may tend to perform poorly during Pre-Oxygenation and are less effective, with only 40% oxygen or less being delivered during spontaneous ventilation. This is because the Resuscitation bags using duck-bill inspiratory valves function differently during manual versus spontaneous ventilation [7]. ATMO-Vent is purely based on manual mechanical ventilation, where there is flow only during inspiration as opposed to the continuous flow observed in spontaneous ventilation, and hence the duck-bill valve should not be of concern regarding the Pre-Oxygenation.

### 3.2 Actuating mechanism

The linear actuator is the core of the ventilator. Various modes of actuation have been proposed in various DIY ventilator designs such as gripping arm design of E-Vent [2], compressed air in OxVent [3], and Rack and pinion mechanism in ApolloBVM [4]. The use of an electric linear actuator in ATMO-Vent provides a versatile approach to control the stroke length and stroke speed, which makes it possible to control the frequency of breaths and tidal volume, both parameters that are monitored and are adjustable in commercial mechanical ventilators. This controlled motion allows for a continuous volume of air resulting in a typical breathing cycle and highly customizable for patient’s age and size. In contrast, other actuating mechanisms like the automated single-direction camming solution do not allow versatile control of the volume of air as needed for a typical breath cycle. The linear actuators used in ATMO-Vent do not involve the use of a feedback circuit and use built-in limit switches to detect the position of the actuator. The use of external sensors has not been considered as 1) it makes the design more complicated involving more sensors and increasing risks of the failure, and 2) cost of the development increases with the additional sensors. Hence, the displacement of the linear actuator in ATMO-Vent is controlled through an open loop system using PWM signals.

The choice of the linear actuator is one of the most critical design factors to consider in building ATMO-Vent. ATMO-Vent uses an adult-sized BVM which has a height of approximately 120 mm in the distended state. BVM, when operated by hand, provides a volume of approximately 500 ml per squeeze. The tidal volume into the lung represents the average volume of air displaced between normal inhalation and exhalation when extra effort is not applied. It is approximately calculated by multiplying the bodyweight of the patient in kg, times 6 ml per kg. [8]. In any case, the choice of TV is decided by the respiratory therapist and is an important parameter to be controlled and monitored. The stroke length decides the tidal volume delivered to the patient. The frequency of breath is another crucial parameter to be controlled, and patients with respiratory illness may demand a higher frequency of breaths. The stroke length and speed decide the maximum stroke speed of the linear actuator limits the minute volume delivered to the patient since the maximum frequency of breaths for a particular tidal volume that can be provided by the ventilator. The current linear actuator LD3 used in ATMO-Vent can provide a maximum stroke speed of 25 mm/s, and this constrains the frequency of breaths in high tidal volume requirements. The 12 V LD3 linear actuator has an inbuilt limit switch that switches off the motor when the end effector is in the highest position or vice-versa. The stroke speed is controlled by VNH3SP30 motor driver module using Pulse Width Modulation (PWM). The master Arduino Mega 2560 generates the PWM signals to control the linear actuator. The Arduino Mega 2560 is chosen for its higher programmable memory of 256 KiloBytes (KB). The software algorithm is discussed in the Computing and Interface section.

### 3.3 Measurements

Pneumatic systems, including life-saving ventilators, need to have a strict measurement system to monitor and control the flow of gases. Ventilators have three basic parameters to be controlled and monitored – Pressure, flow rate and volume. ATMO-Vent uses an Arduino Mega 2560 as a slave microcontroller to monitor pressure and flowrate. The volume of the air-oxygen mixture is calculated from the latter by integrating the flowrate over the inhalation time period. The native 10-bit Analog to Digital Conversion (ADC) capability of Arduino Mega 2560 limits the resolution of the output voltages measured from the Pressure and flowrate sensors. This is mitigated by using an external 16-bit ADC such as ADS1115 interfaced to the Arduino using an I^2^C interface. The ADS1115 is a four-channel programmable gain amplifier with gain up to 16x. A gain of 1x is used with the pressure and flow measurements since the output voltages from the sensors are in the range of the 5.0 V reference voltage used.

#### 3.3.1 Pressure sensing and control

The pressure is an important parameter to be controlled as it is commonly associated with Ventilator Induced Lung Injury (VILI) such as barotrauma. Excess pressure can be fatal to the patient by damaging the alveoli, or an under-pressure may not be able to overcome the resistance of the patient’s lungs preventing the lungs from getting the required airflow. Hence, there needs to be tight monitoring and control of the pressure. SDP2000-L Differential Pressure sensor from Sensiron is used in ATMO-Vent connected to the proximal tube. The sensor has been designed specifically for ventilators, and its range of −100 Pa to 3500 Pa best fits the pressure monitoring requirements. The low limit (-100 Pa) of the sensor can provide a negative pressure trigger capability of 1 cm H_2_0. The other pressure-based measurands such as the Peak Inspiratory Pressure (PIP), Mean airway pressure, Plateau Pressure, Positive End-Expiratory Pressure (PEEP) are calculated from the pressure measured. The sensor operates at 5 V and provides a 0.25–4.0 V analogue output with zero offset and excellent long-term stability. The sensor is interfaced to the Arduino Mega 2560 via I^2^C through a 16-bit ADS115 breakout board from Adafruit. Eq. (1) is used to convert the raw ADC output of the differential pressure sensor into the pressure values in cm H_2_0.

(1) $$Pressure\left[cmH_{2}0\right]=\left[\left(\left(adc_{value}x0.000125\right)-0.250\right)x3500/3.750\right]x0.0101972$$

A solenoid operating Normally Closed (NC) valve is connected to the proximal tube of ATMO-Vent, to release the excess pressure, in case the pressure generated from the BVM exceeds the PIP set by the healthcare professional. The solenoid valve is connected to the Arduino Mega 2560 through an IRF520 Metal Oxide Field Effect Transistor (MOSFET). The power MOSFET is used to switch a 12 V DC supply to the solenoid valve when the pressure measured exceeds the PIP.

#### 3.3.2 Flow rate measurement

**Fig. 2:**
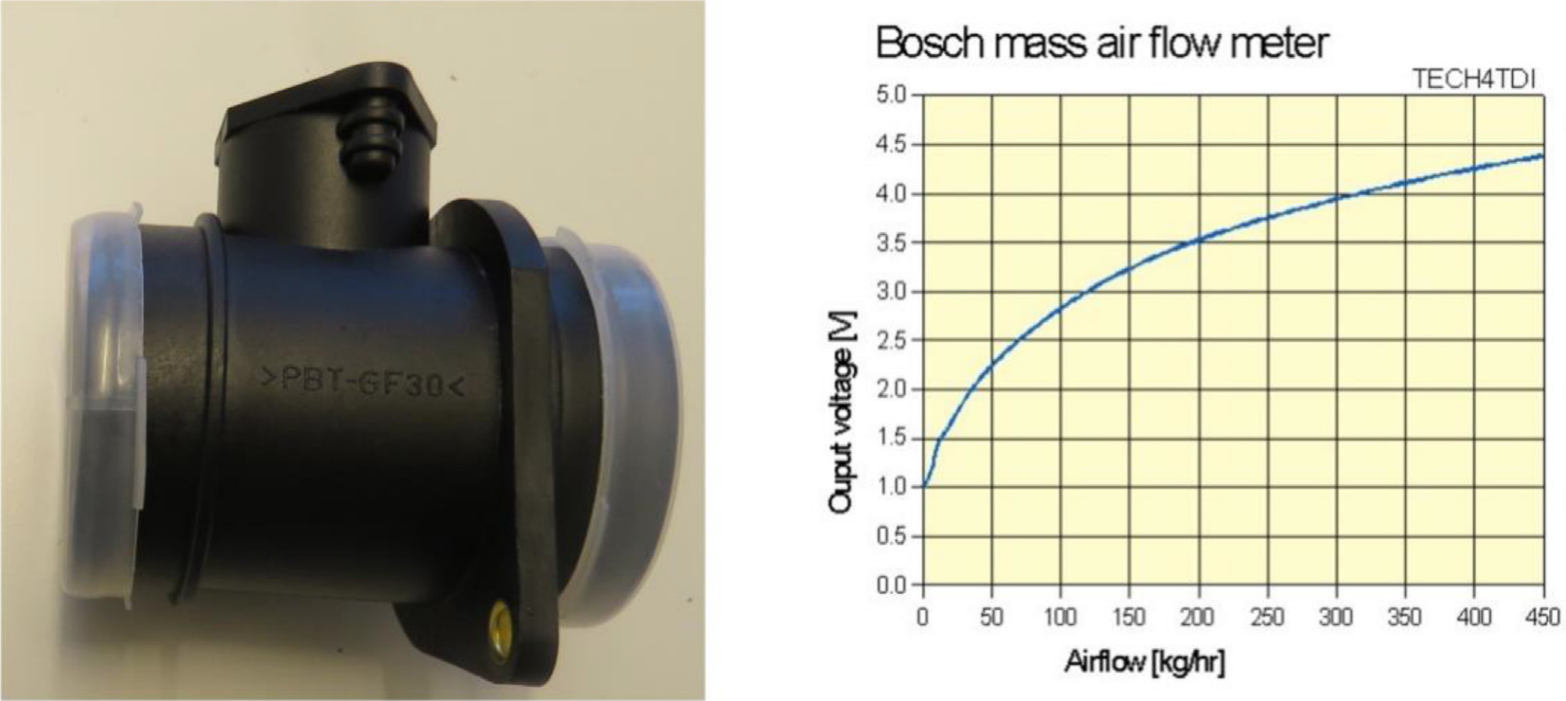
(Left) Mass Air Flow (MAF) sensor in the original casing (Right) Calibration curve for the Bosch MAF sensor.

Flow rate is also a critical parameter to be monitored as it is also associated with VILI. A higher flow rate leads to frictional losses occurring along the airway epithelium leading to tissue damage [9]. Modern Intensive Care Unit (ICU) ventilators provide a flow rate varying between 50 LPM to 60 LPM. Due to the unavailability, during the pandemic situation, of a commercial flow rate sensor with this lower range of flow measurement apart from the sensors specifically manufactured for ventilators such as Sensiron SFM3000, we explored new possibilities that can be used for DIY ventilators. Flow rate sensors for air medium operate by the pressure difference across venturimeter, ultrasonic principle, or hot-wire anemometer. The hot-wire anemometer method is widely used in automobile engines as a Mass Air Flow (MAF) sensor. The robust, low-cost, efficient and readily available MAF sensor is chosen for ATMO-Vent. The MAF sensors have a very high measurement range of flow up to few thousand kg/hr, while the small petrol engine cars with TDIH engines having the least range of 480 kg/hr. Moreover, they cannot be directly interfaced to the 18 mm or 30 mm flex hoses used in BVM, owing to their larger diameter. Hence, a custom-made 3D-printed part (Flow Meter Adaptor) was designed for ATMO-Vent to couple the MAF sensor to the existing 18 mm flex hose used in ATMO-Vent. The sensor operates at 12 V and provides a 0–5 V analogue output depending on the flow rate. Fig. 2 (Left) shows the MAF sensor core in the inlet manifold tube and a calibration curve of the MAF sensor used in ATMO-Vent is shown in Fig. 2 (Right).

**Fig. 3:**
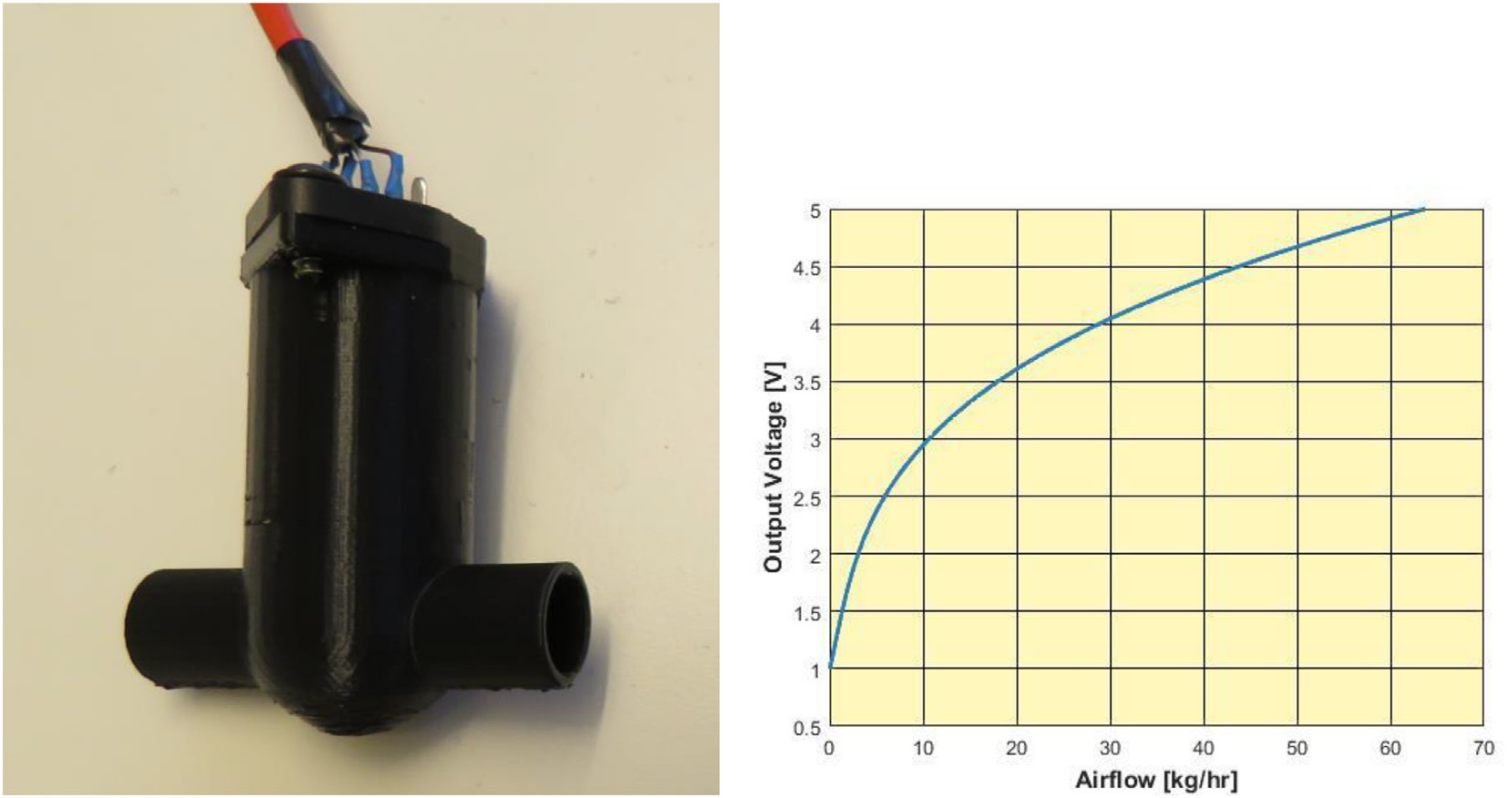
(Left) 3D printed MAF sensor holder with the MAF sensor core attached (Right) Calibration curve in accordance with the 3D printed MAF sensor holder.

This commercial calibration curve provided by the manufacturer cannot be directly used to calibrate the MAF sensor, as they have been calibrated for the larger diameter engine inlet manifold. The calibration curve has been scaled down to match with the diameter of the 3D printed component holding the MAF sensor core. Fig. 3 (Left) shows the MAF sensor core attached to the 3D printed MAF core holder and Fig. 3 (Right) shows the scaled-down calibration curve. Eqs. (2), (3), (4), (5) are used to convert and calculate the scaled-down calibration curve. The calculation has been done in MATLAB code, which is also attached to the article.

(2) $${Flowrate}_{1}\left[m^{3}/s\right]={Airflow}_{1}\left[kg/hr\right]/\left(1.29*3600\right)$$

(3) $${Flowvelocity}_{1}\left[m/s\right]={Flowrate}_{1}\left[m^{3}/s\right]/\left(pi*0.062^{2}\right)$$

(4) $${Flowrate}_{2}\left[m^{3}/s\right]={Flowvelocity}_{2}\left[m/s\right]*\left(pi*0.018^{2}\right)$$

(5) $${Airflow}_{2}\left[kg/hr\right]={Flowrate}_{2}\left[m^{3}/s\right]*1.29*3600$$

here subscript ‘1’ denotes the original engine inlet manifold, and ‘2′ denotes the adapted configuration with the 3D printed MAF sensor holder for ATMO-Vent.

The output voltage of the flow meter is obtained from the raw ADC measurements using the Eq. (6).

(6) $$Voltage\left[V\right]=adc_{\mathrm{value}}x0.000125$$

The calibration function between the measured output voltage and the equivalent airflow is given by Eq. (7). The coefficients are obtained by curve fitting with a R^2^ value of 0.9476.

(7) $$Airflow\left[kg/hr\right]=11.9782*{Voltage}^{3}+44.8774*{Voltage}^{2}+86.7963*Voltage-53.8707$$

**Fig. 4:**
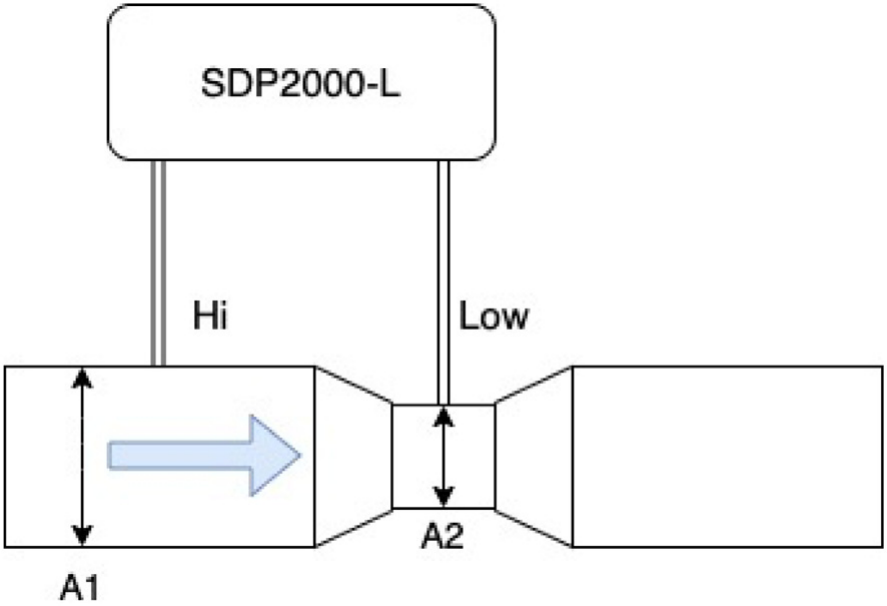
Differential Pressure sensor-based flow meter.

As an alternate method, the flow rate can also be calculated using a venturimeter with a differential pressure sensor. The pressure drop between the entrance and the throat of the venturimeter provides the flow rate. The SDP2000-L differential pressure sensor is again used for this venturimeter. The block diagram of the venturimeter is shown in Fig. 4.

The flow rate is calculated from the raw ADC output of the SDP2000-L pressure sensor as follows using Eqs. (8) and (9):

(8) $$deltaP\left[N/m^{2}\right]=\left[\left(\left(adc_{value}x0.000125\right)-0.250\right)x3500/3.750\right]$$

$$Flowrate\left(LPM\right)=A_{2}x\sqrt{\frac{2xdeltaP}{\left(\left(rho*\left(1-{\left(\frac{A_{2}}{A_{1}}\right)}^{2}\right)\right)\right)}}x60000$$

where A_1_ is the Area of the entrance, A_2_ is the Area of the throat, and rho is the density of air = 1.225 kg/m^3^. The area A_1_ and A_2_ affect the maximum flow rate that can be measured with the venturimeter.

The MAF sensor is the preferred flow meter owing to its higher accuracy and a high-frequency response allowing measurement of turbulent flows. In either case, the sensor is connected to the output of the BVM between the first and the second duck-bill valve such that they measure the inspiration flow rate. Either sensor can detect a reverse flow when placed along the proximal tube, but the measurements would not be accurate, and hence it is placed in the inhalation phase.

#### 3.3.3 Volume determination

The volume of the air-oxygen mixture flowing out of the BVM is calculated from the flow rate measurements by integrating the flow rate over the inspiration time. The volume calculation is done on the master Arduino Mega 2560 of ATMO-Vent. The volume measured is sent to the python software running on the Raspberry Pi. Raspberry Pi 4 Model B 2 GB RAM is used in ATMO-Vent for its higher processing speed of 1.5 GHz with a Quad-core Cortex-A72 (ARM v8). The Quad-core design allows multi-threading of the ATMO-Vent software. The software architecture is detailed under the Computing and Interface section. The volume calculation yields TV, Minute Ventilation, Resistance and Compliance.

### 3.4 Power supply and conditioning

Most of the components of ATMO-Vent operate at 12 VDC except for the Raspberry Pi, that operates at 5 VDC. The most robust, cost-effective and readily available power supply module was from the Desktop Personal Computer (PC) itself. An ATX CPU power supply is used in ATMO-Vent with a maximum power output of 320 W. The power supply provides 12 VDC with a peak current capability of 16 A, which is more than sufficient to power the modules of ATMO-Vent which consumes 44 W. M185A, a robust automotive variable DC-DC converter from Kemo Electronics, is used in ATMO-Vent to step down the 12 VDC voltage to 5 VDC needed by Raspberry Pi.

### 3.5 Computing and interface

**Fig. 5:**
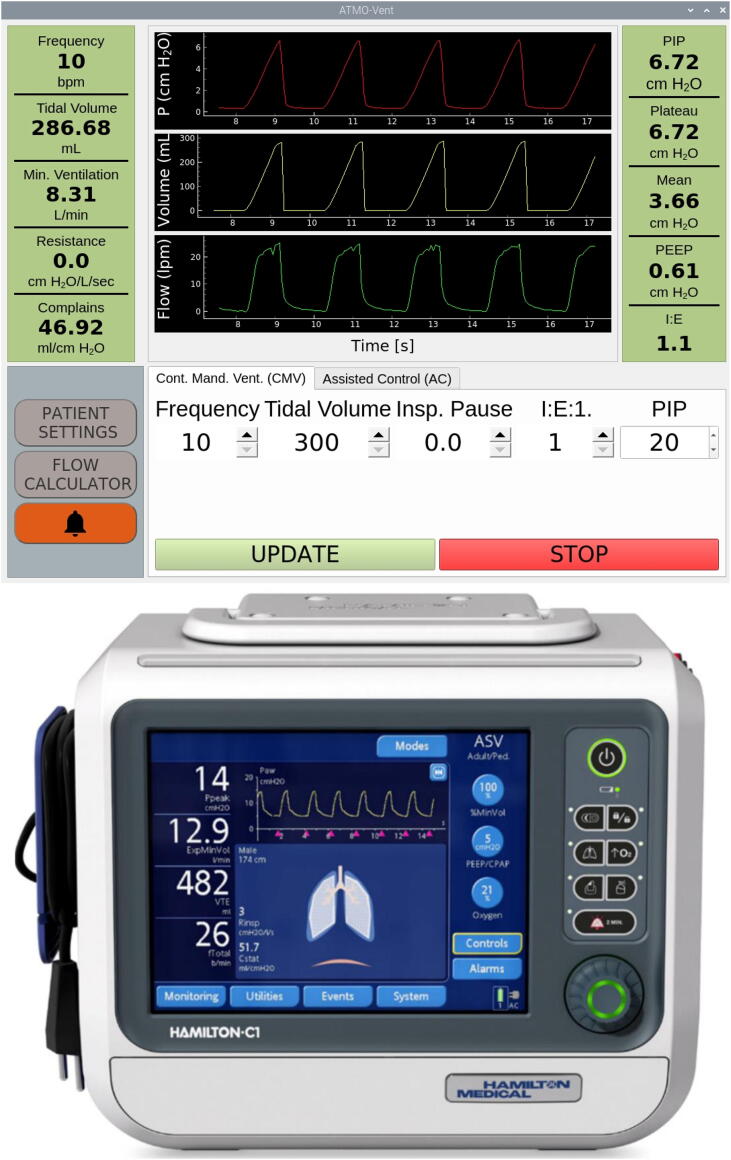
(Above) GUI of ATMO-Vent with the respiratory parameters on either side in the green background and the settings panel in grey at the bottom. The pressure, flow rate and volume measurements are displayed as real-time plots. (Below) Hamilton-C1 monitor showing essential respiration parameters. (Credits: www.hamilton-medical.com) This GUI model has been used as a reference for the ATMO-Vent GUI design. (For interpretation of the references to colour in this figure legend, the reader is referred to the web version of this article.)

This section describes the software architecture of ATMO-Vent, which provides the feedback and control of ATMO-Vent. The processing unit of ATMO-Vent consists of two Arduinos and the Raspberry Pi computer. Raspbian operating system is used on the Raspberry Pi and it could be replaced with Realtime Operating Systems (RTOS) as the program itself has been designed in python to be flexible with a multitude of operating systems. The two Arduinos are connected in a master-slave SPI configuration with the Arduino Mega 2560 constituting the master Arduino connected to the linear actuator VNHCP30 motor driver and the IRF520 power MOSFET. The slave Arduino Mega 2560 is connected to a 16-bit ADC breakout board, Adafruit ADS1115. The two Arduinos are connected through a serial interface to the Raspberry Pi 4 operating at a baud rate of 115,200 Kbps. The GUI of ATMO-Vent is designed in python3 with graphical widgets for control, in a way very similar to commercial ventilators, as shown in Fig. 5.

**Fig. 6:**
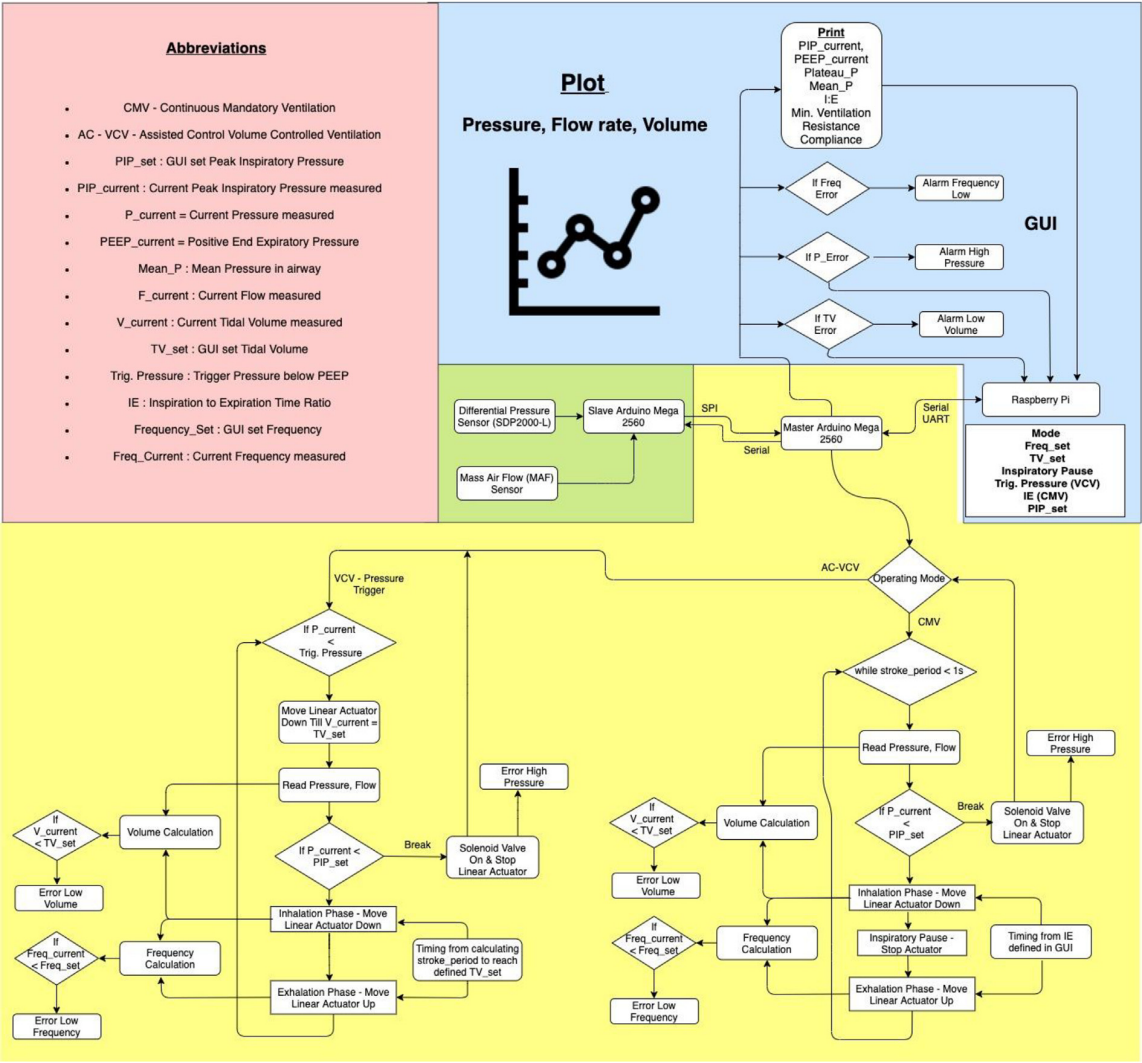
ATMO-Vent software architecture sub-divided in three levels, where the green zone represents the slave Arduino, yellow represents the master Arduino and Blue represents the Raspberry Pi. The abbreviation labels are explained in the pink section, on the top left. (For interpretation of the references to colour in this figure legend, the reader is referred to the web version of this article.)

A touch-based desktop monitor can be connected to ATMO-Vent via an HDMI connector to control the GUI. A keyboard and monitor can also be used with an ordinary monitor if the former is not available. A buzzer is also integrated into the General-Purpose Input-Output (GPIO) of the Raspberry Pi. The buzzer alarms whenever crucial parameters such as PIP, frequency and TV are out of range from the set values. Fig. 6 shows the software architecture of ATMO-Vent with a consolidated flowchart.

The master Arduino Mega 2560 (Yellow section) receives the operation command from the GUI through serial communication and performs the inhalation and exhalation sequence based upon the requested mode. It also reads the pressure and the MAF sensor flow data in real-time from the slave Arduino Mega 2560 (Green portion) through an interlinked Serial – SPI interface. The master Arduino Mega 2560 then calculates the TV, the inhalation time and exhalation time, frequency and other respiratory parameters such as the PIP, Plateau Pressure, and PEEP based upon the input settings. The two Arduinos work in tandem with the Raspberry Pi, and through efficient thread management, a smooth GUI interaction is guaranteed. The GUI displays the respiratory parameters measured in real-time and also calculates the resistance and compliance, which are used by the healthcare professionals to monitor patients on the ventilator and determine the lung recovery. The modes of operation are detailed in the Operation Instructions section of the article.

### 3.6 PC cabinet

An ATX Desktop PC cabinet has been modified to enclose the ventilator. The main reason to enclose all the hardware in a PC cabinet casing is to comply with the Portable Appliance Testing (PAT) standards stated in the UK Guidelines for RMVS. The guidelines mention compliance with the IEC 60601, IEC 62,353 standards, which emphasizes on Electro-Magnetic Compatibility (EMC). In addition to protecting the elements inside from ambient exposure in the clinical environment (for example to liquids, dust, or undesired human contact), the PC cabinet shields off electromagnetic noises and also provides immunity to the electronics against Electro-Magnetic Interference (EMI). The PC cabinet fans also help to regulate the temperature of the Raspberry Pi computer and prevents over heating with continuous operation. ATMO-Vent has undergone rigorous testing and qualifies under Class B requirements of EN 55,011 CISPR 11 standards. The testing procedure is detailed in the Validation and Testing section of the article.

Overall, this low-cost, rapidly developable ATMO-Vent ventilator would support the patients with respiratory distress by:

• Providing invasive ventilation by continuous mandatory ventilation for patients with ARDS.
• Providing a non-invasive assisted ventilation support to patients who need not have to be intubated.
• Providing monitoring of the critical respiratory parameters for diagnosis.

## 4 Design files

The source to the design files can be found in the Mendeley repository > Data > Design Files.

**Table:** 

Design file name	File type	Open source license	Location of the file
Pressure Sensor & Relief Adaptor	.STP, .STL	CC BY 4.0	Mendeley > Data > Design Files
MAF Sensor Adaptor	.STP, .STL	CC BY 4.0	Mendeley > Data > Design Files
BVM Holder Input End	.STP, .STL	CC BY 4.0	Mendeley > Data > Design Files
BVM Holder Output End	.STP, .STL	CC BY 4.0	Mendeley > Data > Design Files
BVM Piston	.STP, .STL	CC BY 4.0	Mendeley > Data > Design Files
MR3000 Flow Meter Fixture	.STP, .STL	CC BY 4.0	Mendeley > Data > Design Files
Duck-bill Valve Adaptor	.STP, .STL	CC BY 4.0	Mendeley > Data > Design Files
ARDUINO_MASTER	.INO	GNU General Public License (GPL) v3	Mendeley > Data > Arduino Software
ARDUINO_SLAVE	.INO	GNU General Public License (GPL) v3	Mendeley > Data > Arduino Software
ATMO_RPI_GUI	.py	GNU General Public License (GPL) v3	Mendeley > Data > GUI Software
FLOW_METER_CALIBRATION	.m	GNU General Public License (GPL) v3	Mendeley > Data > Flow Meter Calibration
FLOW_METER_CALIBRATION_DATA	.txt	CC BY 4.0	Mendeley > Data > Flow Meter Calibration > Sample
Quick guide for building ATMO-Vent	.docx	CC BY 4.0	Mendeley > Data > Quick Guide

Pressure Sensor and Relief Adaptor is the CAD file of the 3D printed sensor and valve hose adaptor.

MAF Sensor Adaptor is the CAD file of the 3D printed adaptor housing the MAF sensor.

BVM Holder Input End is the CAD file of the 3D printed front holder to hold BVM.

BVM Holder Output End is the CAD file of the 3D printed rear holder to hold BVM.

BVM Piston is the CAD file of the 3D printed piston that is attached to the linear actuator.

MR3000 Flow Meter Fixture is the CAD file to cut the aluminium sheet to fix the MR3000 flow meters.

ARDUINO_MASTER is the source code to the master Arduino Mega 2560.

ARDUINO_SLAVE is the source code to the slave Arduino Uno.

ATMO_RPI_GUI is the python3 source code of the ATMO-Vent GUI.

FLOW_METER_CALIBRATION is the MATLAB code to obtain the calibration curve for the MAF sensor with the 3D printed adaptor.

FLOW_METER_CALIBRATION_DATA is the data used to obtain the calibration curve for the MAF sensor.

Quick guide for building ATMO-Vent, is the document which summarizes the steps to build ATMO-Vent.

All the CAD files can be 3D printed with PLA material [10] using a 0.8 mm nozzle with a layer height of 0.2 mm. The “Pressure Sensor & Relief Adaptor” part may require using a 0.4 mm nozzle to print the smaller pressure sensing tubes in the design correctly. The “MR3000 Flow Meter Fixture” could ideally be cut in a 1.2 mm thick aluminium sheet, but it may also be 3D printed.

## 5 Bill of Materials

The full bill of materials is shown in Table 1. The table is subdivided into six categories with different colour coding, as mentioned in the hardware description section. The source links to the components can be found in the Bill of Materials (BOM) excel document uploaded to the Mendeley repository > Data > Bill of Materials.

**Table 1:** Bill of materials of ATMO-vent.

Total cost: GBP 1000 approx.

## 6 Build Instructions

This five-step instruction guide provides a visual overview of the assembly actions required to build an ATMO-Vent unit. Each component has been assigned a part code with the nomenclature in the Bill of Materials. A quick guide for building ATMO-Vent is also enclosed in the Mendeley repository.

**Fig. 7:**
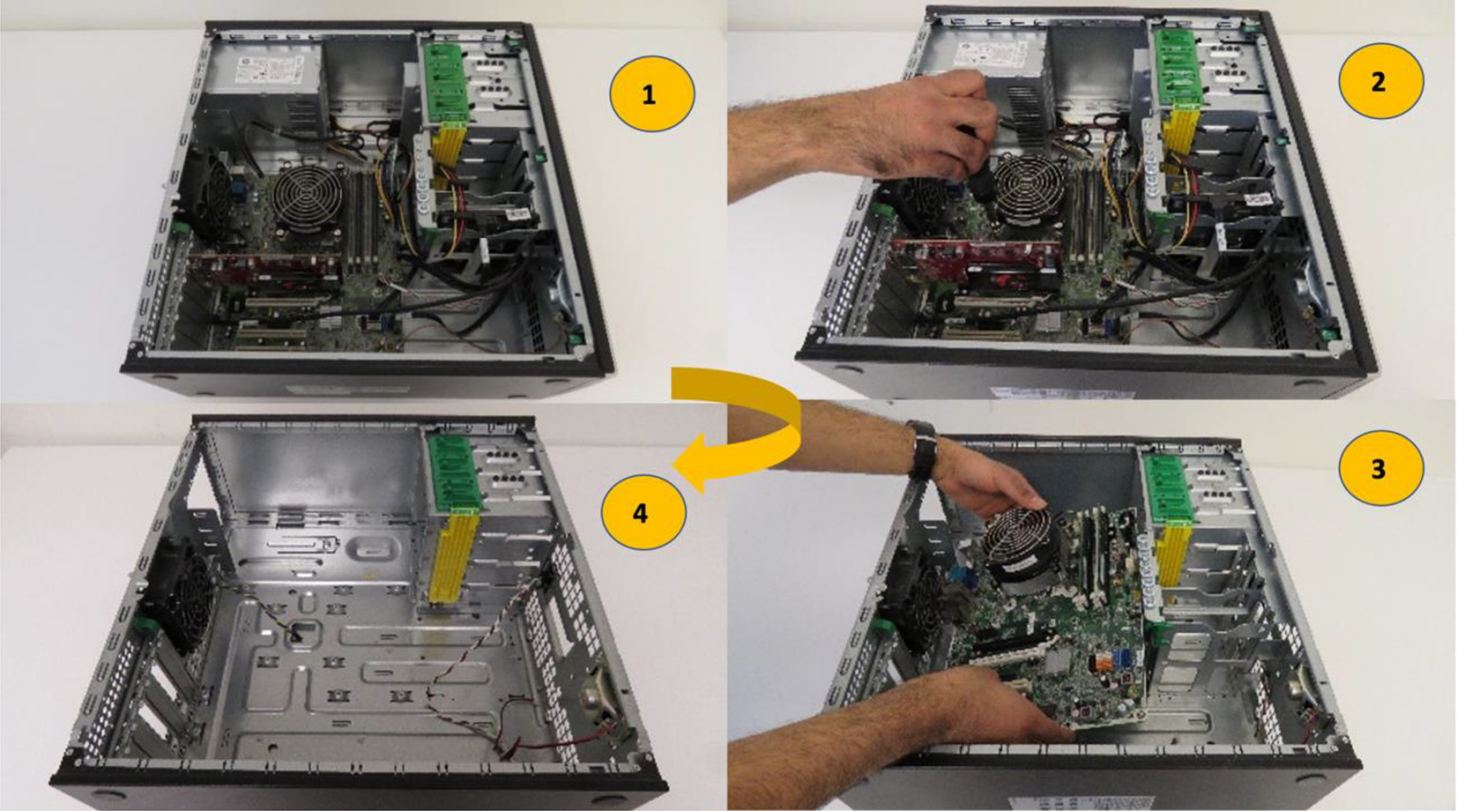
The first step of ATMO-Vent building – PC Cabinet preparation.
Step 2 (Fig. 8): Dismantle the CD/DVD drive and remove the CD tray and the underlying electronics. Use the metal enclosure to house the ATMO-Vent electronics. The two Arduino Mega 2560 (E1) with the 16-bit ADS1115 ADC shield (C3), and VNHSP30 motor driver (B2) are mounted inside the CD drive using the double-sided tape (F7), and proper cabling is done using the shielded CAT5 cable (E4), shielded twisted pair cable (E5) and jumper wires (E7) fitted with headers (E6) on the ends. The ground in the shielded cables are soldered to the base of the CD drive enclosure to provide a single point grounding. This is crucial to reduce the noise generated by the electronics. Use adhesive copper tape (F10) to wrap any spaces in the CD drive enclosure to make a perfect faraday cage. The CAT5 cable (E4) connects the ADC shield (C3) input to the MAF sensor.

Step 1 (Fig. 7): Prepare the PC Cabinet (F8) by removing the motherboard and hard disk bay. The hard disk bay is secured to the base of the Cabinet using eyelets. Use a drill with a diameter similar to the eyelet head to remove the eyelet. Exercise caution and use Personal Protection Equipment (PPE), especially for the eyes, when performing this step as tiny metal fragments can be released during this operation. Replace the cabinet power switch with an ON-OFF switch.

**Fig. 8:**
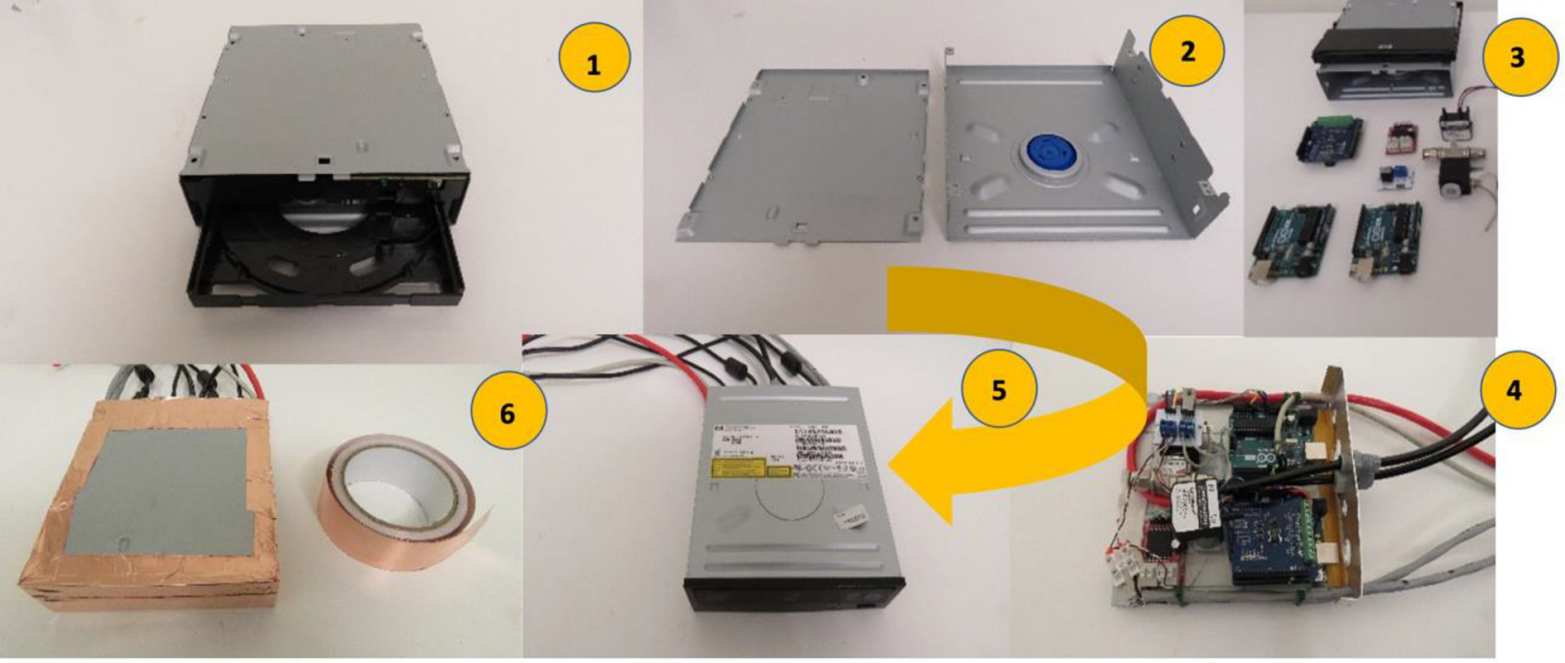
Second step of ATMO-Vent building – Electronics assembly.
Step 3 (Fig. 9): The 1.2 mm aluminium plate (F4) is cut, as shown in 1 (top left) of Fig. 9 as per the CAD design of the part “MR3000 Flow Meter Fixture” provided in the Mendeley repository. Flowmeters (A4) are attached to the aluminium plate. Use proper PPE when working with the aluminium sheet. Chamfer the edges to prevent sharp edges. The 8 mm tubing (A1) is used to route the input from the air/oxygen inlets to the inlet of the flowmeters through the NPT 1/8 threaded connector (A3). The output of the flowmeter is connected to individual non-return check valves (A5). The check valves are, in turn, connected to the Y-branch (A6). The union of the Y-branch is connected to the oxygen inlet of the BVM (A7) through the same 8 mm tubing (A1). The parts mentioned above are enclosed inside the PC cabinet. The tubing outside the PC cabinet involves the “Duck-bill Valve 2” where the PEEP valve assembly adaptor (A8) is connected for exhaust.

**Fig. 9:**
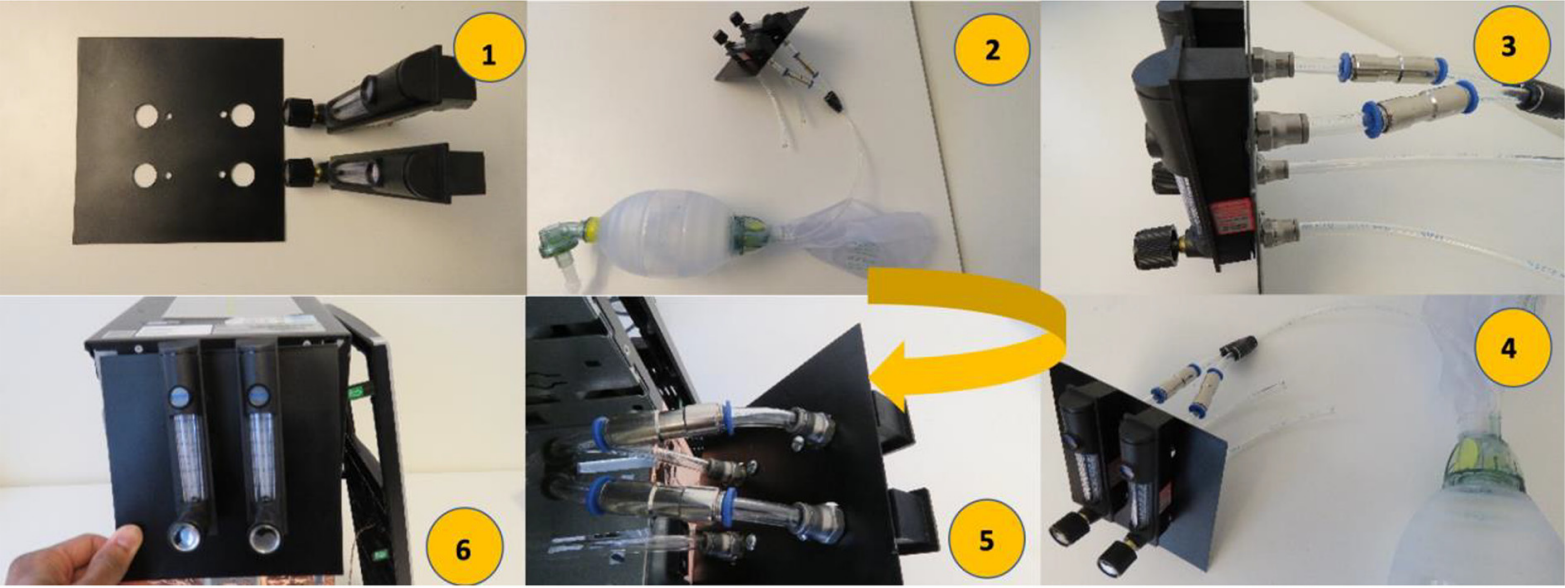
The third step of ATMO-Vent building – Air-Oxygen mixture circuit.
Step 4 **(**Fig. 10**):** Use the 2 mm aluminium sheet (F3) to prepare a base for the linear actuator assembly. The aluminium needs to be cut as per the profile in the PC cabinet where the motherboard used to be fastened. Chamfer the edges to avoid sharp edges. Attach the linear actuator (B1) to the aluminium profile (F1) through the fixtures provided with the actuator. Fasten the aluminium profile to the sheet (F3) using T-bolts (F2). Use the same screws used to fasten the motherboard to fasten the sheet (F3) to the base of the cabinet. Fix the power supply unit (D2) to the cabinet and fasten with screws. Fig. 10 (top) shows the components before assembly into the PC cabinet. The six modules follow the same colour code nomenclature, as indicated at the beginning of the article. Fig. 10 (bottom) shows the modules assembled in the PC cabinet. The 3D printed “MAF Sensor Adaptor” is fixed right after the “Duck-bill Valve 1” assembly attached to the outlet of the BVM inside the PC cabinet. The extra hoses (F9) are used to connect the 3D printed “Pressure Sensor & Relief Adaptor” outside the PC Cabinet after the “Duck-bill Valve 2” assembly as close as possible to the patient. “Duck-bill Valve 2” is obtained by removing the right-angled outlet port from another BVM and connected to the flex hose through the 3D printed Duck-bill Valve Adaptor. 6 mm Tubing (A2) is used to connect the 3D printed “Pressure Sensor & Relief Adaptor” outlets to the Differential Pressure sensor (C2) and solenoid valve (C5) respectively. This tube, after the “Duck-bill Valve 2” assembly, is called the proximal tube. This tube is attached to the patient through a face mask or endotracheal tube. The proximal tube assembly is shown in Fig. 10 (Bottom).

**Fig. 10:**
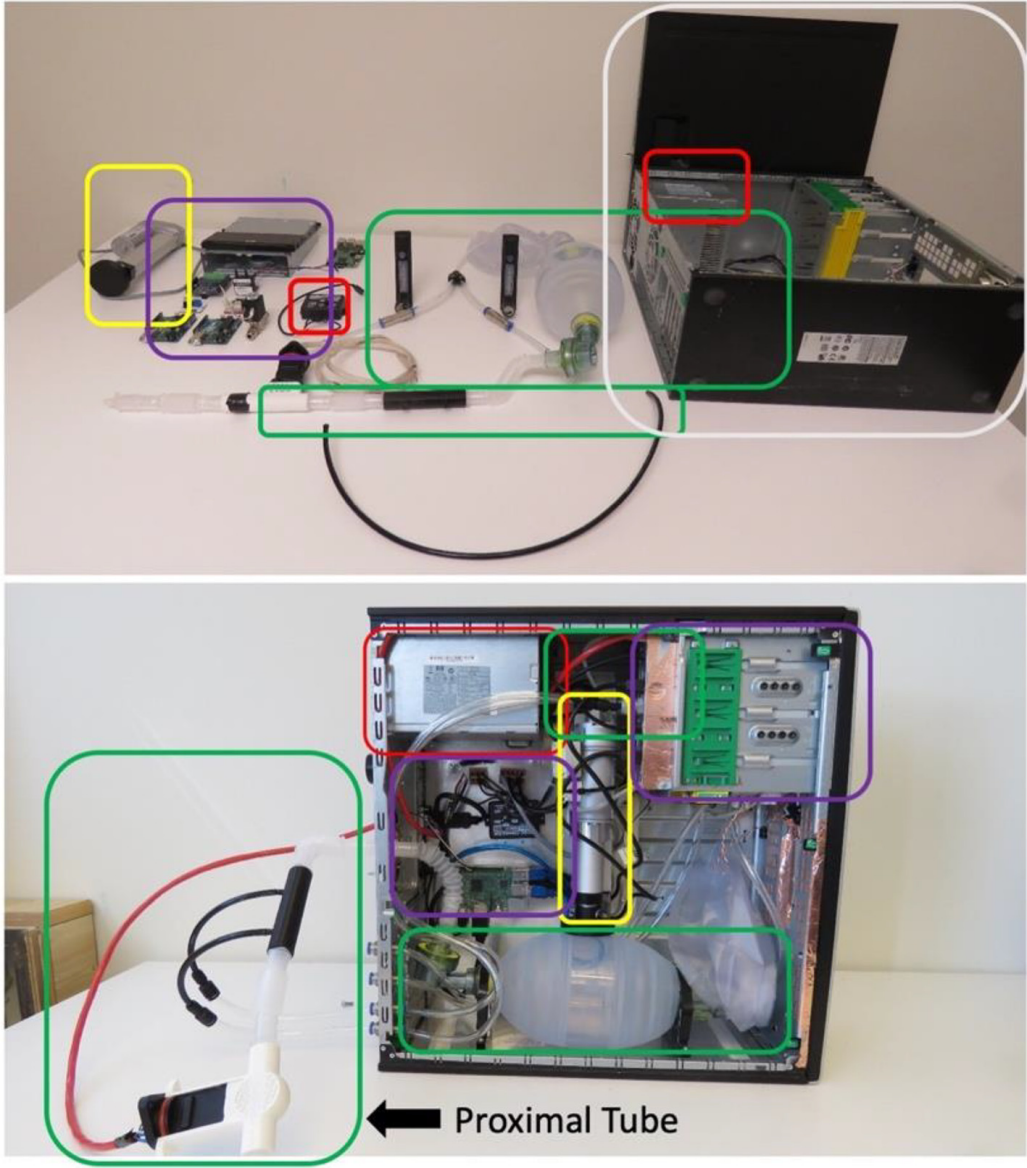
The fourth step of ATMO-Vent building – Assembling the individual sub-modules.

The Raspberry Pi computer (E2) enclosed in its casing is secured to the aluminium sheet (F3) using double-sided tape (F7). The existing PC cabinet rear panel mount is used to fix the panel mount adaptors – HDMI (E11) and USB (E8). The adaptors are connected to the Raspberry Pi through the respective cables (E1011). Power cabling from the PC power supply is routed through Terminal Block connectors (D3, D4, D5 and D6). It should be ensured that the Raspberry Pi is connected to a 5 V supply of DC-DC converter through a micro USB port (E12) and not to the 12 V input supply from the PC power supply. The ON-OFF switch is connected to the ground and switch wire of the PC Power supply. The switch wire can be found from the datasheet of the power supply.

**Fig. 11:**
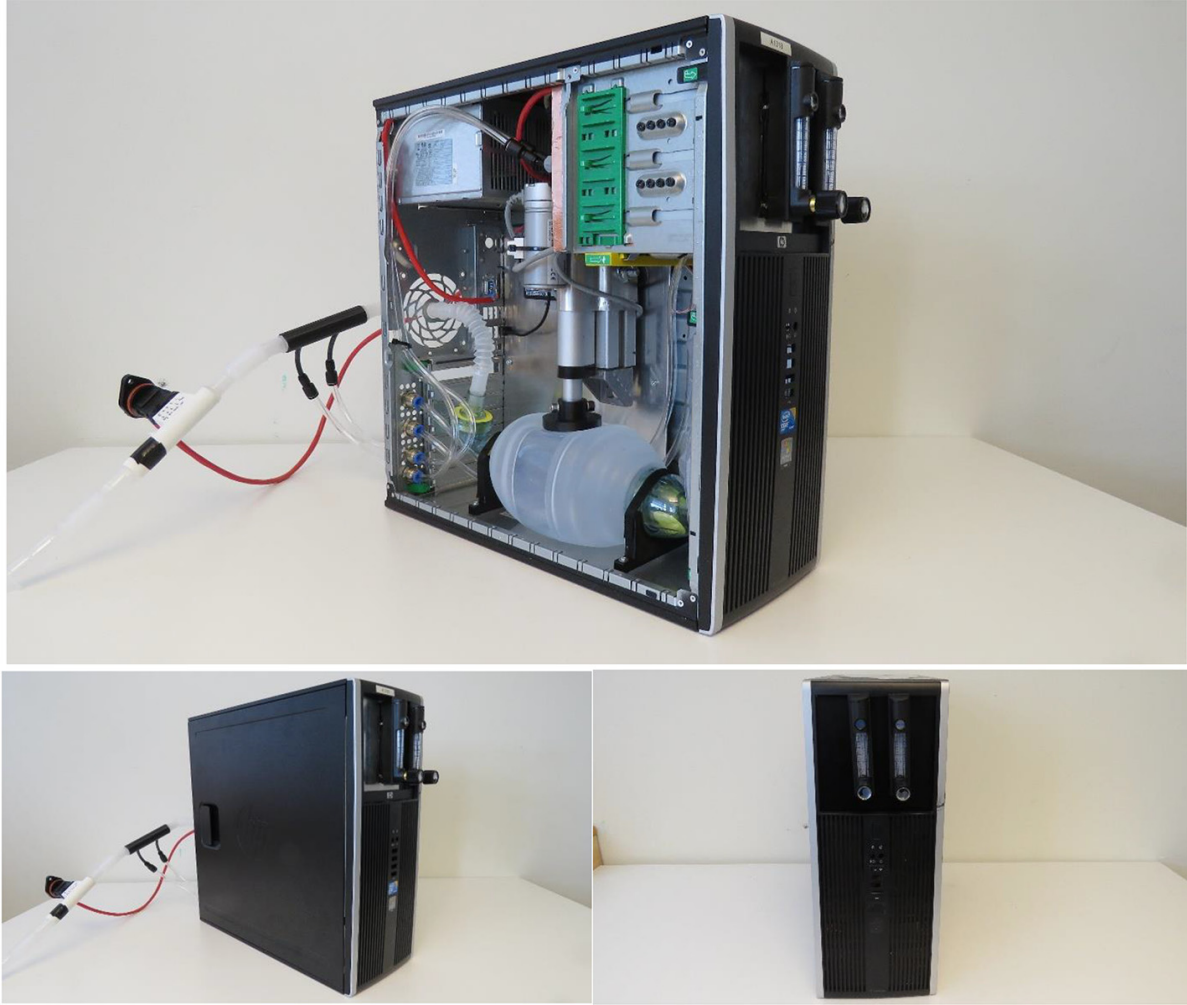
The final step of ATMO-Vent building.

Step 5 (Fig. 11): With all the components connected, the PC cabinet side door is fixed and secured using its screws. The ATMO-Vent is now ready for operation. Fig. 11 (top) shows the fully assembled ATMO-Vent with side door opened and Fig. 11 (bottom) shows the side and front view of ATMO-Vent with the side door fixed.

## 7 Operation instructions

ATMO-Vent operation is very straight-forward, and the GUI is very intuitive, resembling the modern mechanical ventilators. ATMO-Vent has two modes of ventilation – Continuous Mandatory Ventilation (CMV) and Assisted Control Ventilation (AC). Both modes follow Volume Control Ventilation (VCV), where tidal volume is the primary regulating parameter. The following steps outline the operation of ATMO-Vent.

Step- IX: Set the PEEP pressure by rotating the knob on the spring-loaded PEEP valve. Note down the PEEP setting as this would be used in the next step to define the negative trigger pressure.
Step – X: In the case of AC ventilation, select the Assisted Control Ventilation Tab. Enter the minimum frequency of breaths, desired TV in ml, Inspiratory Pause (0.0 to 0.5) in seconds, and Trigger Pressure. The Trigger Pressure depends upon the PEEP settings. Ideally, without any PEEP settings, the default Trigger Pressure is −1 cm H_2_0. With a PEEP setting, e.g. 5 cm H_2_0, the trigger pressure has to be set as 4 cm H_2_0.

**Fig. 18:**
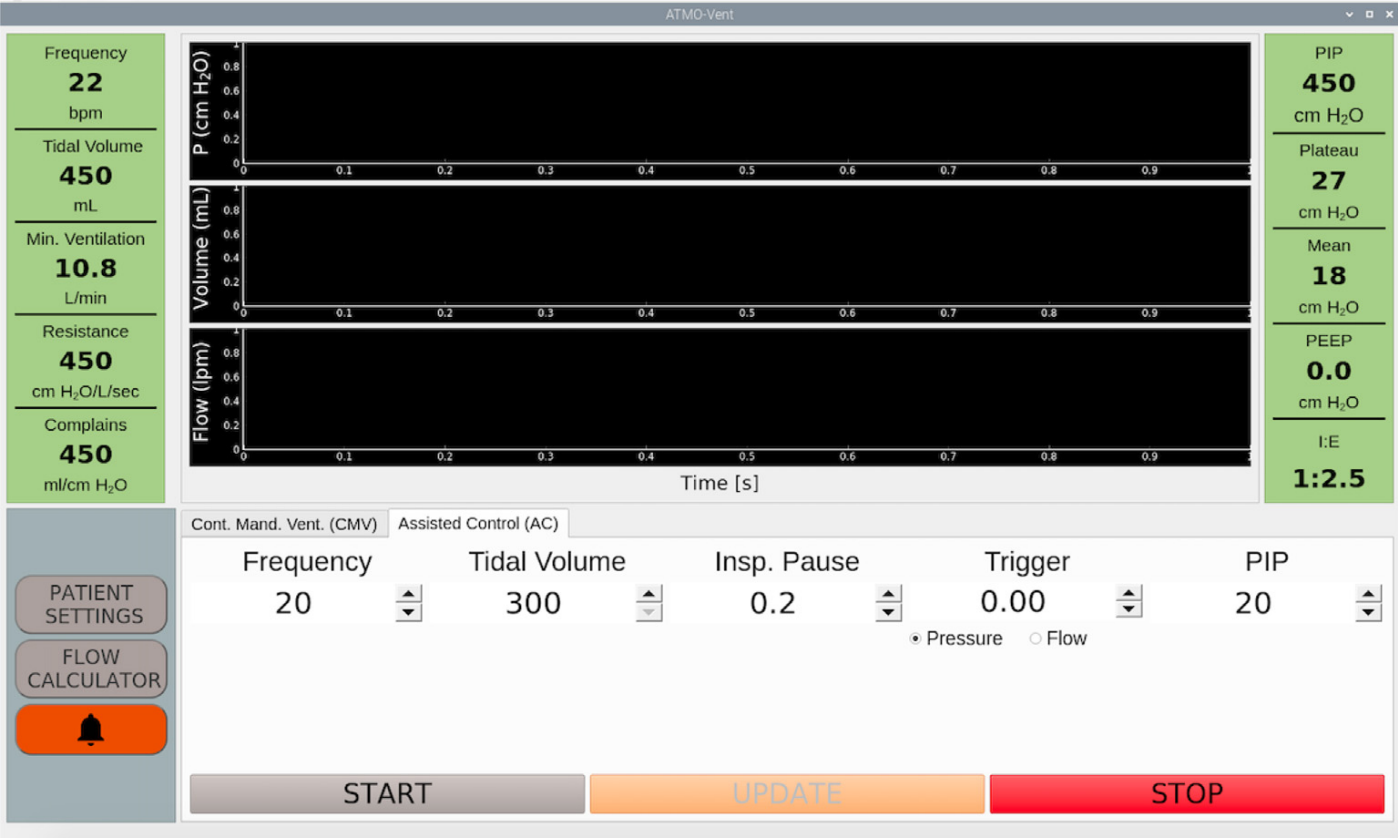
AC Mode GUI with parameters filled in.

Then enter the Peak Inspiratory Pressure (PIP) value in the corresponding textbox in units of cm H_2_0. The PIP value determines the maximum allowed inspiratory pressure into the airway. All the values must be entered in the respective text boxes and double-checked before proceeding to Step XII. Fig. 18 shows the GUI with all the values filled in for the AC Mode.

**Fig. 19:**
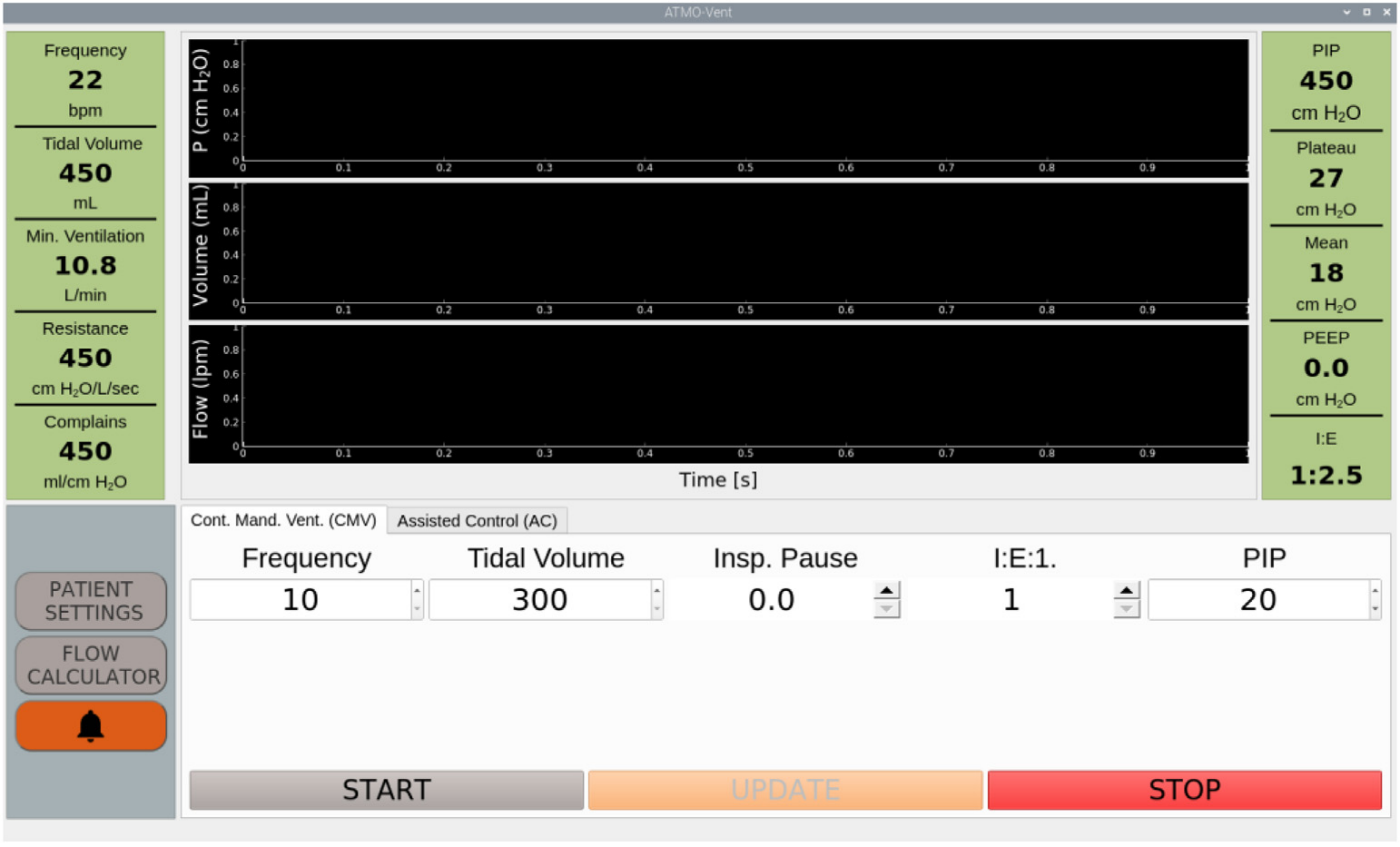
CMV Mode GUI with parameters filled in.
Step – XII: This step is more of a medical procedure and is out of the scope of this article. In the case of NPPV, fasten the BVM mask on the patient and ensure that it has a tight seal around the patients’ nose and mouth. In the case of invasive ventilation, intubation has to be performed by a trained healthcare professional. Once the necessary procedure is done, it must be ensured that the proximal tubing is securely connected to the patient.
Step – XIII: With the proximal tube double-checked in place, the start button on the GUI is pressed. In the case of CMV mode, the ventilator would start immediately and ventilate the person. In case of Assisted control modes, the ventilator would wait for the patient to trigger the inhalation sequence and then would ventilate the patient. Modifications to the respiration parameters is allowed during the ventilator operation after the Start button is pressed, by entering the new parameters and pressing the Update button on the GUI.

Step – XI: In the case of CMV ventilation, select the Continuous Mandatory Ventilation Tab. The settings of CMV are very similar to AC ventilation except for the Trigger settings. CMV is a mandatory mode where the ventilator provides inhalation and exhalation sequence with the programmed frequency, tidal volume, Inspiratory pause, I/E setting and maximum PIP that can be encountered. In CMV mode, the maximum inspiration time is by default restrained to one second. Thus, the downward operation of the linear actuator will occur until the set tidal volume is reached or for one second, whichever precedes. All the values must be entered in the respective text boxes and double-checked before proceeding to Step XII. Fig. 19 shows the GUI with all the values filled in for CMV mode.

### 7.1 Alarms

ATMO-Vent has three crucial alarm features respective to CMV or AC mode, namely PIP alarm, frequency alarm and tidal volume alarm.

#### 7.1.1 PIP alarm

This alarm fires in both CMV and AC mode, when the PIP pressure exceeds the maximum PIP set by the healthcare professional. The label with the increased PIP value would highlight in red, accompanied by a high-frequency buzzer. The increased PIP value would quickly revert to the healthcare professional set maximum PIP value, as the linear actuator would immediately cease motion and safety solenoid valves would open. The buzzer can only be turned off after the acknowledgement from the operating physician by clicking the bell button.

#### 7.1.2 Frequency alarm

This alarm fires only in the AC mode, when the patient has breaths per minute below the minimal value set by the healthcare professional. This alarm is indicated by the low bpm value highlighted in red, accompanied by the high-frequency buzzer sound. The healthcare professional has to manually turn off the alarm by clicking the bell button and decide further course of action on the patient.

#### 7.1.3 Volume alarm

This alarm can fire in both CMV and AC mode. ATMO-Vent being a volume-controlled ventilator is driven by tidal volume as the primary control variable. There can be scenarios when the needed tidal volume is not being delivered to the patient. This can be due to any leaks in the proximal tube or increased fluid accumulation in the lungs. The alarm is indicated by the low tidal volume highlighted in red, and the buzzer is producing a high-frequency buzzing sound. Again, this alarm can only be turned off manually by a healthcare professional by clicking the bell button after assessing the situation.

## 8 Validation and characterization

ATMO-Vent, similar to any medical device, has to be approved by any public body. Several critical factors have to be validated and characterized. This section discusses the compatibility of ATMO-vent in terms of its performance and compliance with the RMVS guidelines of UK-MHRA. The Electromagnetic Compliance (EMC) and Electromagnetic Interference (EMI) tests have been conducted in accordance with the IEC 60601: Medical design standards for power supplies, which is one of the minimum criteria to be satisfied according to the RMVS guidelines by UK-MHRA.

### 8.1 Performance characteristics:

**Fig. 20:**
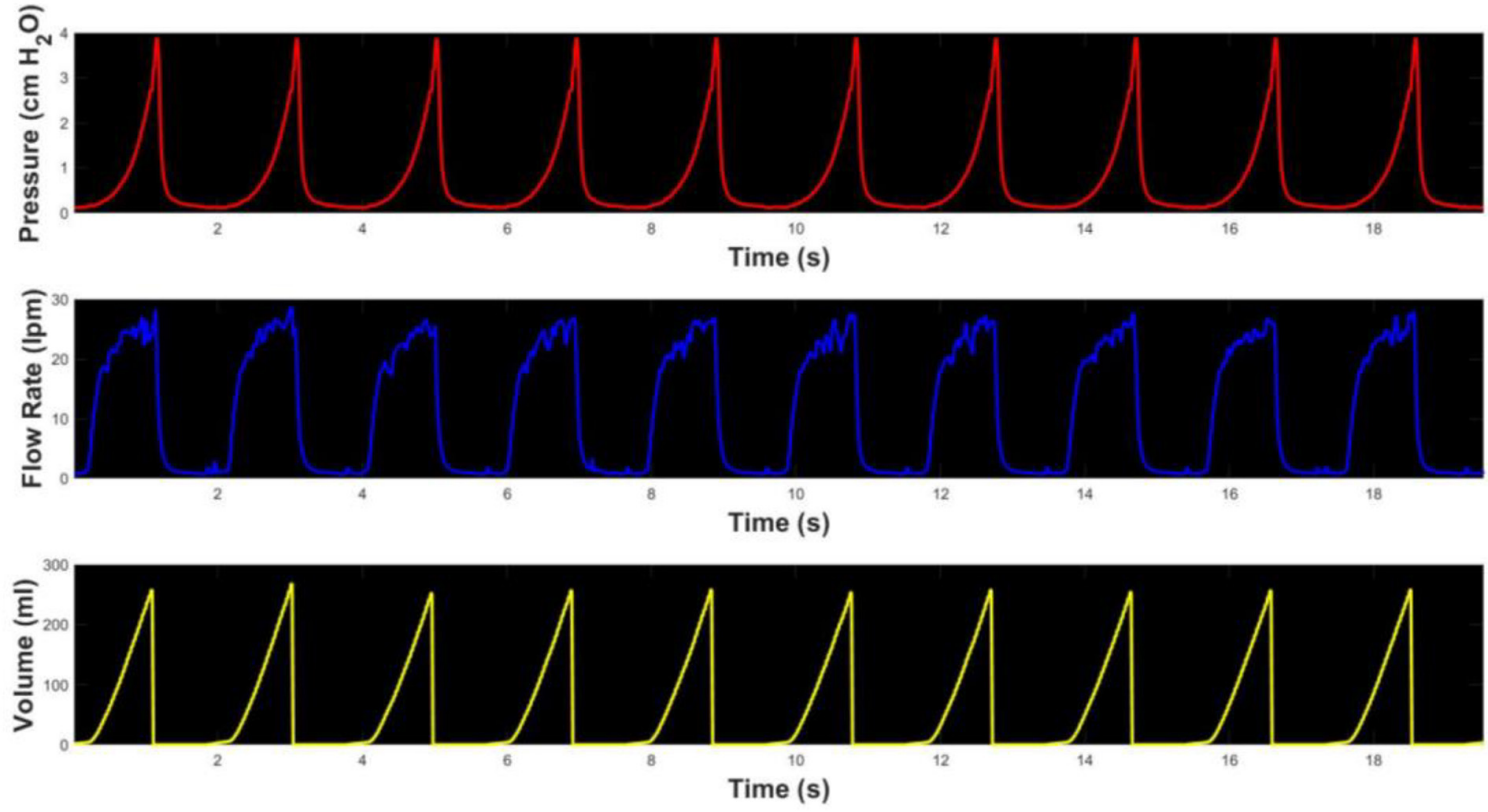
Pressure, flow rate and volume plots during 10 continuous breaths in CMV mode.

A demonstration scenario with Continuous Mandatory Ventilation (CMV) mode was executed on a Mannequin with the input respiration parameters that resulted in the following output as provided in Table 2. Fig. 20 shows the pressure, flow rate and volume plots during the 10 consecutive breaths during the ATMO-Vent operation.

**Table 2:** Input and output respiration parameters in Continuous Mandatory Ventilation (CMV) mode.

Respiration parameter	Input	Output
Frequency (bpm)	30	29
Tidal Volume (ml)	300	267.7
Inspiration pause (sec)	0	0
I:E ratio	1	1
Peak Inspiratory Pressure (PIP, cm H_2_O)	20 (cut-off pressure)	3.89
Positive End-Expiratory Pressure (PEEP, cm H_2_O)	0	0.11

The achieved frequency and tidal volume (highlighted in red in Table 2) is lower than the set values in the CMV mode. Therefore, the minute volume supplied by ATMO-Vent is lower than the set input value. The reason for this discrepancy is because of the limitation in the speed of the linear actuator. The maximum inspiration time for a patient is one second and the flow rate should be such that the minute volume should be achieved. The lower speed of the current linear actuator limits the minute volume achieved by the reduced flow rate. Typically, a flow rate of 50–60 LPM is required for a patient under ventilation. In the following version of ATMO-Vent, we plan to improve the stroke speed of the linear actuator to attain a higher flow rate and match the required minute volume. Further tests are to be performed with a lung simulator in order to quantify the output respiration parameters accurately.

### 8.2 Patient inflating valve validation

**Fig. 21:**
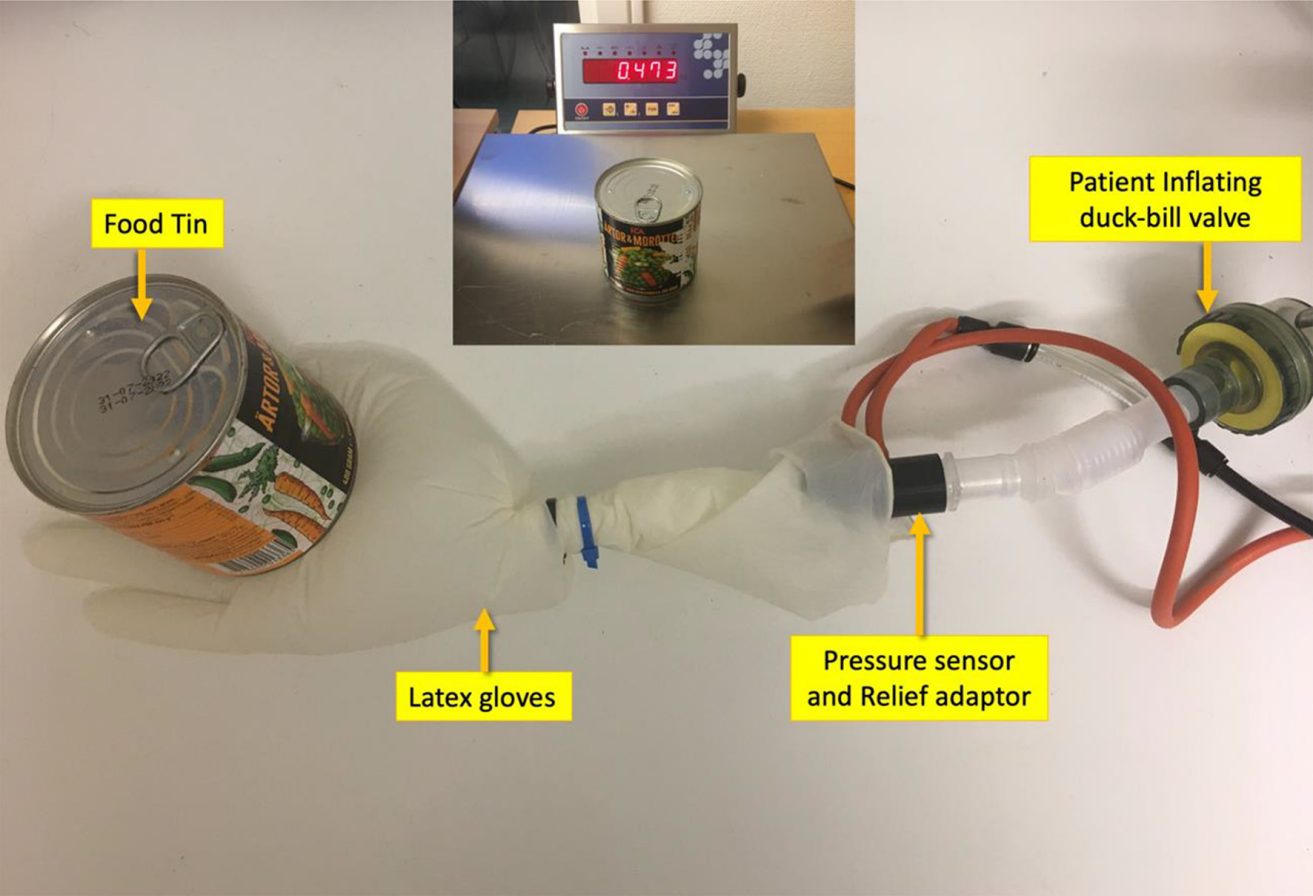
Patient Inflating Valve test using an expandable latex glove acting as a lung and a packaged food tin simulating the lung resistance. The inset image shows the packaged food tin weighed to be 473 g.

The validation of a duck-bill valve as a patient inflating valve is discussed in this section. The second duck-bill valve used in ATMO-Vent along the proximal tube is fundamentally the patient inflating valve. During manual ventilation, the air-oxygen mixture is forced through the valve base, opening the duck-bill valve and air-oxygen mixture is delivered to the patient’s lungs. This force also seals the valve base to the exhalation port preventing the fresh air-oxygen mixture from venting through the exhalation port. During exhalation, the valve base returns to its former position, and, thus, exhaled gases are vented through the exhalation port. To validate the optimal operation of the duck-bill valve in ATMO-Vent, a simple experiment is performed to ensure that the expiratory limb of the exhalation circuit is closed despite higher pressures generated by lung resistance. The experimental setup is shown in Fig. 21 as follows.

A latex, flexible and expandable, glove is attached to ATMO-Vent to act as a lung. A packed food tin weighing 473 g is used as a dead weight and is placed over the glove to simulate the lung resistance. A CMV mode of operation with 400 ml tidal volume and 1:2 I:E ratio is used in the experiment. It could be observed from the experiment that the 473 g tin is lifted during the inspiration phase. This experiment validates the operation of the duck-bill valve as a patient inflating valve in ATMO-Vent. The video of the experiment is enclosed in the Mendeley repository for reference.

### 8.3 UK-MHRA compliance table

The RMVS guidelines from UK-MHRA provides consolidated information regarding the set of features a ventilator for the current pandemic situation needs to satisfy. The guidelines outline the minimum modes of ventilator operation, with requirements on monitoring various respiratory parameters in real-time along with safety constraints. The specifications are the minimal clinically acceptable ventilator configuration that can be used in the current COVID-19 pandemic. A ventilator with lower specifications than mentioned in the RMVS guidelines is unlikely to be approved as it is likely to provide no clinical benefit and might lead to increased harm, which would be unacceptable for clinicians. The RMVS guidelines also mention about the biological safety and infection control of the components used in the ventilator. Any part of the breathing system that may come into contact with the patient’s expired gas must be both single patient use only and labelled with an ISO 7000–1051 mark. They should also have appropriate labelling to ensure that they are either one time use or sterilizable. ATMO-Vent uses a double duck-bill valve design which ensures a minimum dead space and also prevents expired gas from travelling back to the BVM. The proximal tube along with the 3D printed “Pressure Sensor & Relief Adaptor”, HEPA filter, and the PEEP valve needs to be changed from person to person. These components are in direct contact with the patients expired gas and are a biological hazard. They have to be carefully handled and disposed. The tubes connecting the 3D printed “Pressure Sensor & Relief Adaptor” can be sterilized or they can also be replaced. The pressure sensor and solenoid valve are the endpoints the expiratory circuit, and they have to be replaced after every patient.

**Fig. 22:**
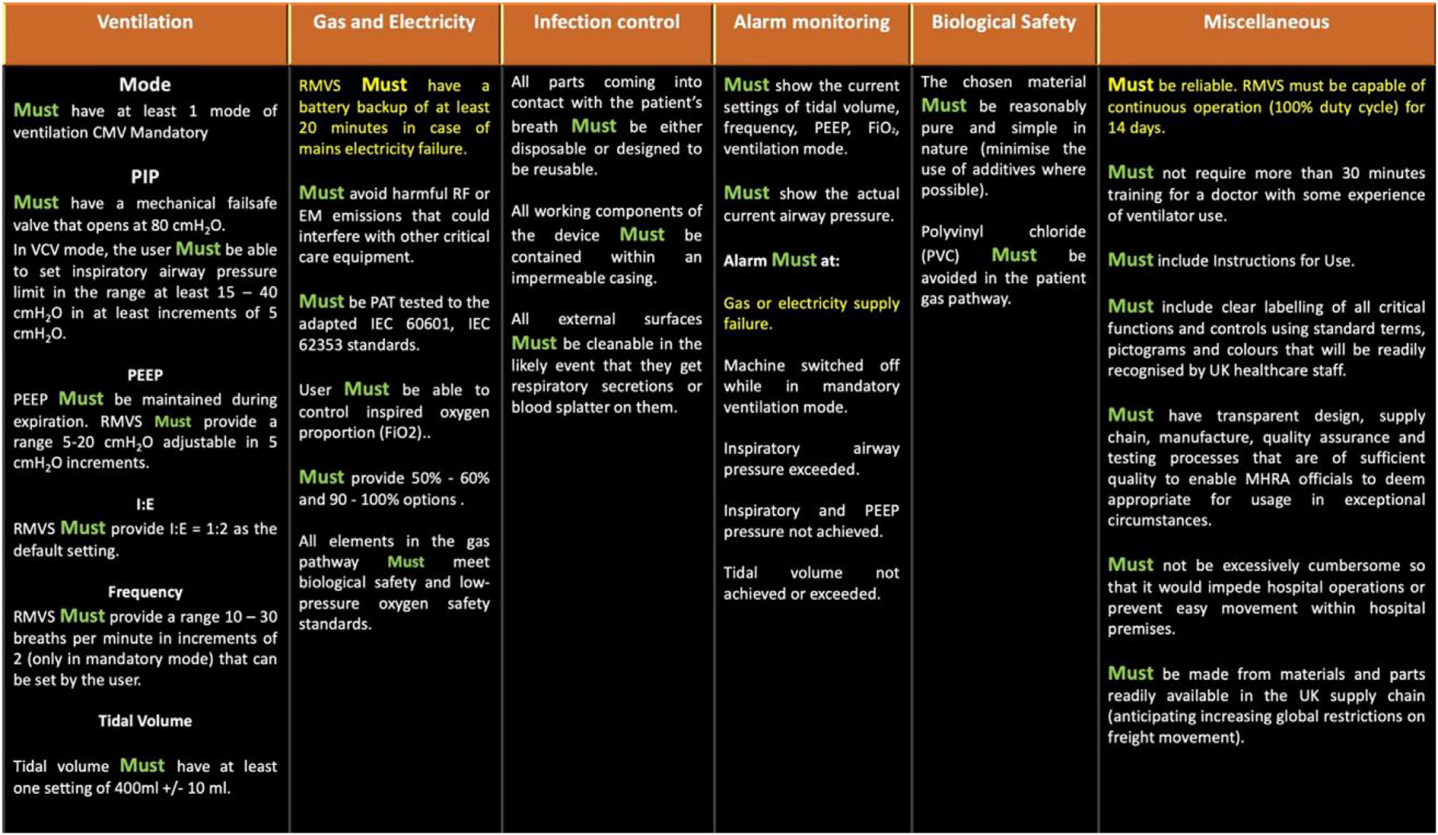
UK-MHRA compliance Table.

A summary of ATMO-Vent compliance with the minimum mandatory requirements of the RMVS guidelines is illustrated in Fig. 22. The text in yellow indicates work in progress for ATMO-Vent. One of the mandatory requirements for a ventilator system is to have a battery backup to keep the ventilator working for a minimum of 20 min until the power supply is restored. ATMO-Vent shall be fitted with a commercial PC Uninterrupted Power Supply (UPS) module, which shall not only provide backup power to the ventilator but also, provide alarms function during electricity failure and also provide shielding to the ATMO-Vent power supply from surges and voltage drops. IEC61000-4-4 electrical fast transient tests, IEC61000-4-5 surge tests, IEC610000-4-6 Conducted RF Immunity test, and IEC61000-4-11 voltage dip, dropout and interruption tests would be performed on the commercial UPS to validate its minimal compliance with the IEC60601 Medical Design Standards for Power Supplies.

### 8.4 EMC/EMI testing

**Fig. 23:**
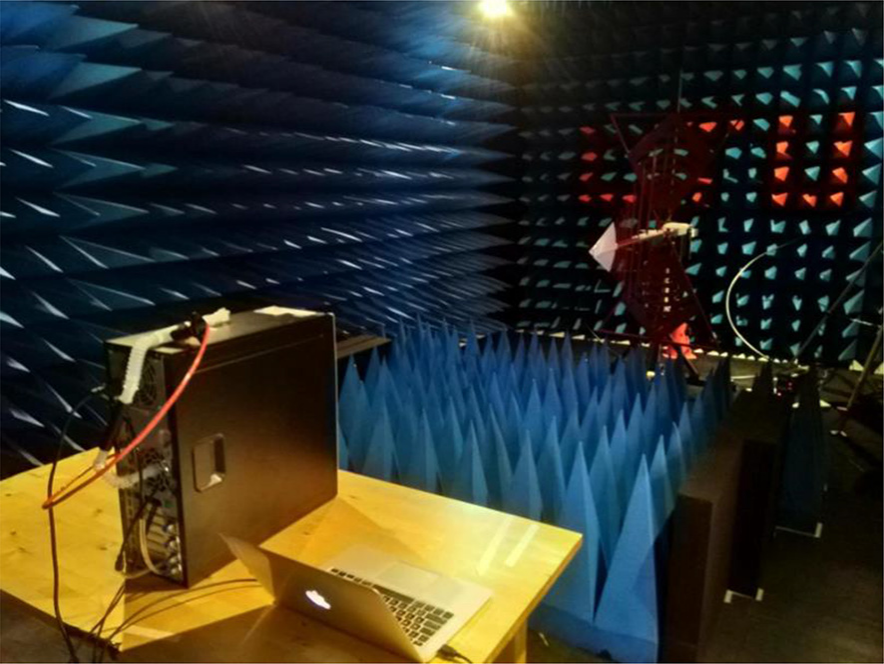
ATMO-Vent undergoing EMC Testing in the Anechoic chamber at Luleå University of Technology, Sweden.

The electromagnetic compatibility (EMC) tests for radiated emission, conducted emission and radiated immunity were so far carried out at the anechoic chamber facility (Fig. 23) at the Luleå University of Technology, Sweden. The series of technical standards encompassed in IEC 60601: Medical design standards for power supplies (document enclosed in Mendeley repository under Data > Medical Standards), were followed to ensure the safety and effectiveness of medical electrical equipment, in our case the ATMO-Vent ventilator. The results for different tests are described below.

#### 8.4.1 Background radiated emission

Test specification: radiated emission EN55022 Class B vertical antenna located at a distance of 3 m from the ventilator. EN55011 Class B device requirements are also in range with the Radiated Emission EN55022 Class B standards.

Scan Settings

**Table:** 

Frequencies			Receiver Settings			
Start	Stop	Step	Res BW	M−Time	Atten	Preamp
30 MHz	1 GHz	40khZ	120 kHz (6 dB)	1 ms	Auto	On

Measurement

**Fig. 24:**
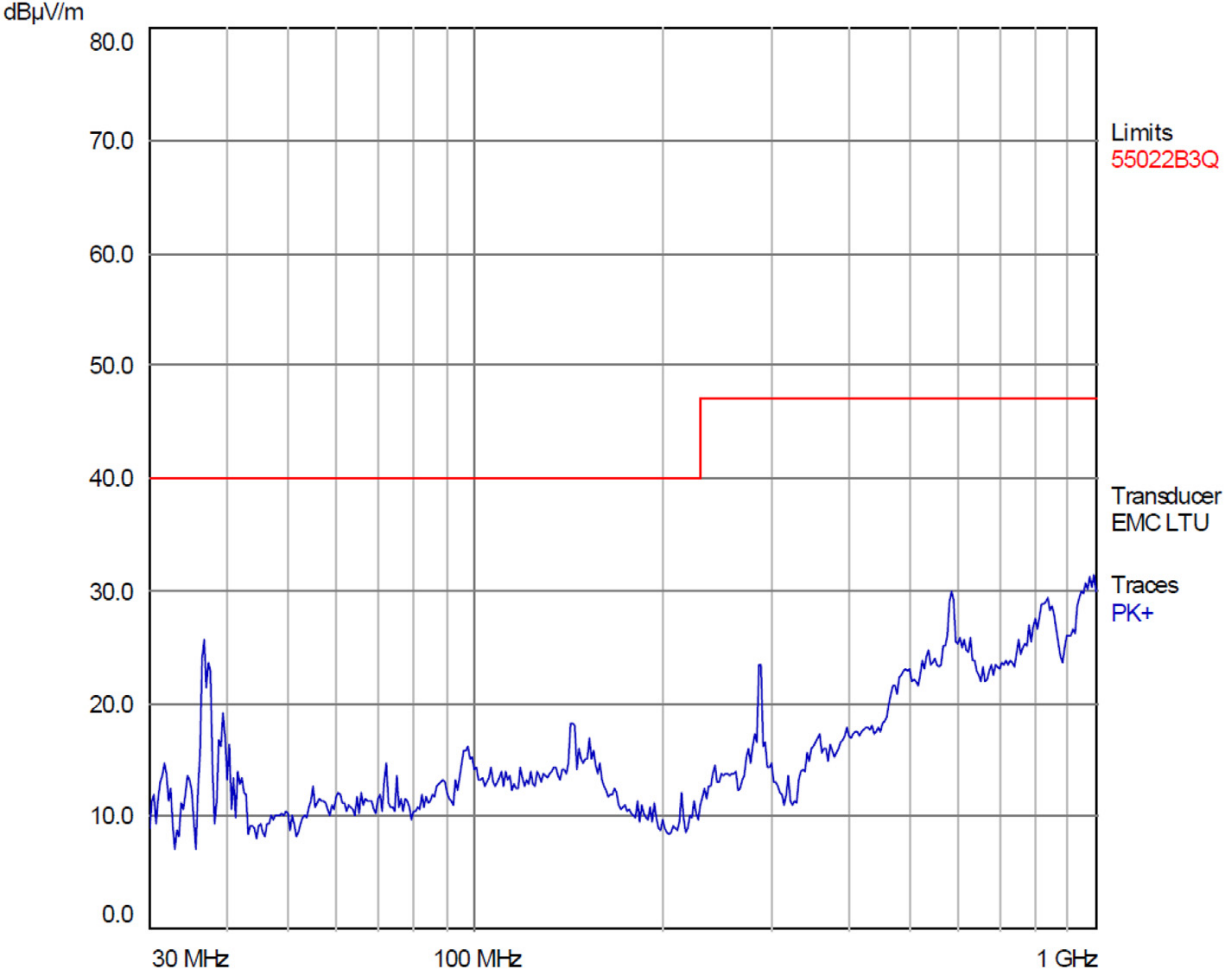
Background radiated emission in the Anechoic chamber.

The background radiated emission level inside the anechoic chamber, where ATMO-Vent was tested for EMC/EMI is shown in Fig. 24.

#### 8.4.2 Radiated emission

Test Specification: Radiated Emission EN55022 Class B Horizontal Antenna located at a distance of 3 m from the ventilator. EN55011 Class B device requirements are also in range with the Radiated Emission EN55022 Class B standards.

Scan Settings:

**Table:** 

Frequencies			Receiver settings			
Start	Stop	Step	Res BW	M−Time	Atten	Preamp
30 MHz	1 GHz	40khZ	120 kHz (6 dB)	1 ms	Auto	On

Measurement

**Fig. 25:**
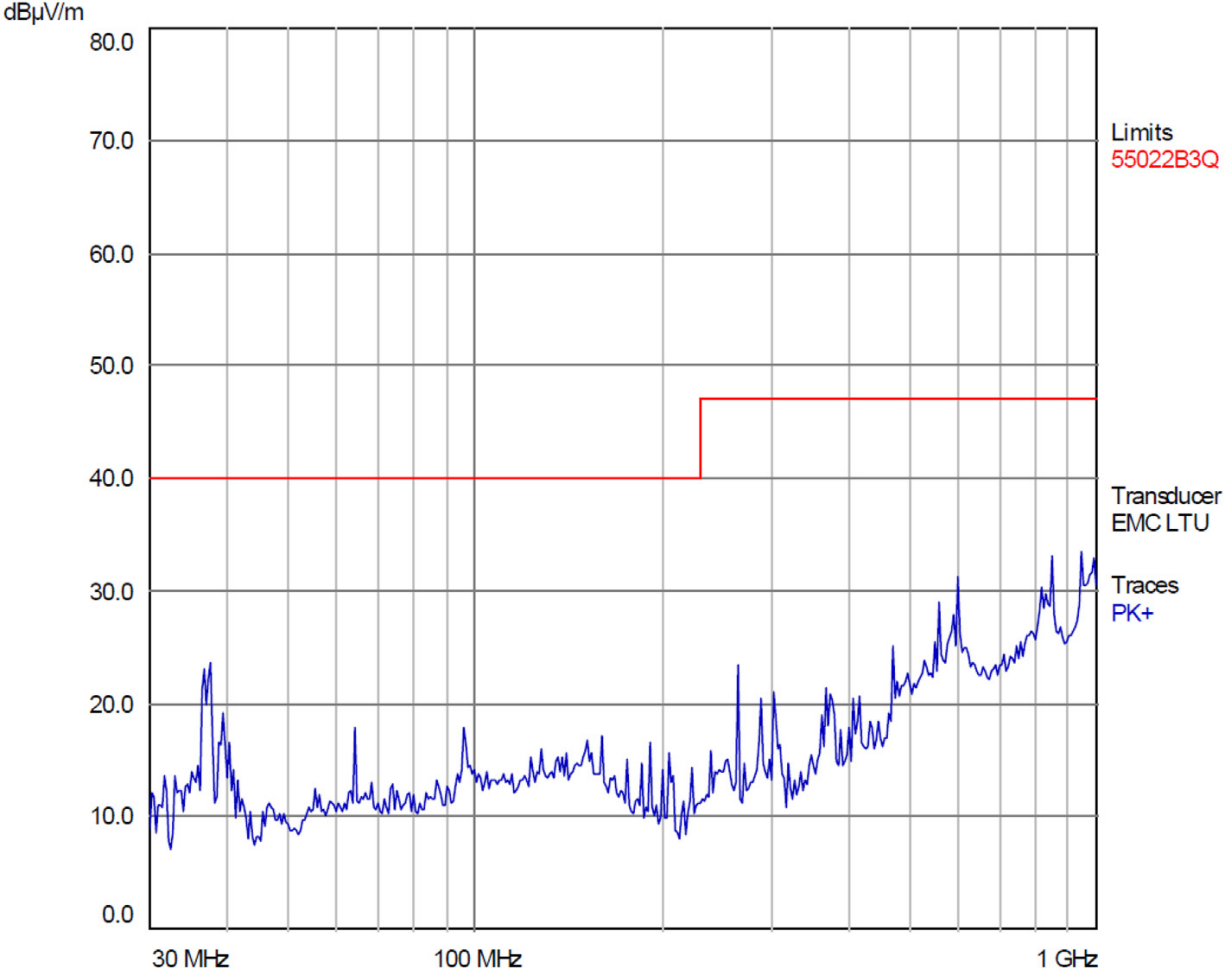
Radiated emission of electromagnetic noises from ATMO-Vent.

The radiated emission tests (Fig. 25) concluded that the ATMO-Vent is well below the 6 dB limit of the range set by the EN55022 Class B requirements.

#### 8.4.3 Conducted emission

Test Specification: EN 55022 Conducted Mains Class B located at a distance of 3 m from the ventilator. EN55011 Class B device requirements are also in range with the Radiated Emission EN55022 Class B standards.

Scan Settings

**Table:** 

Frequencies			Receiver Settings			
Start	Stop	Step	Res BW	M−Time	Atten	Preamp
150 kHz	30 MHz	4.5khZ	9 kHz (6 dB)	10 ms	Auto	Off

Measurement

**Fig. 26:**
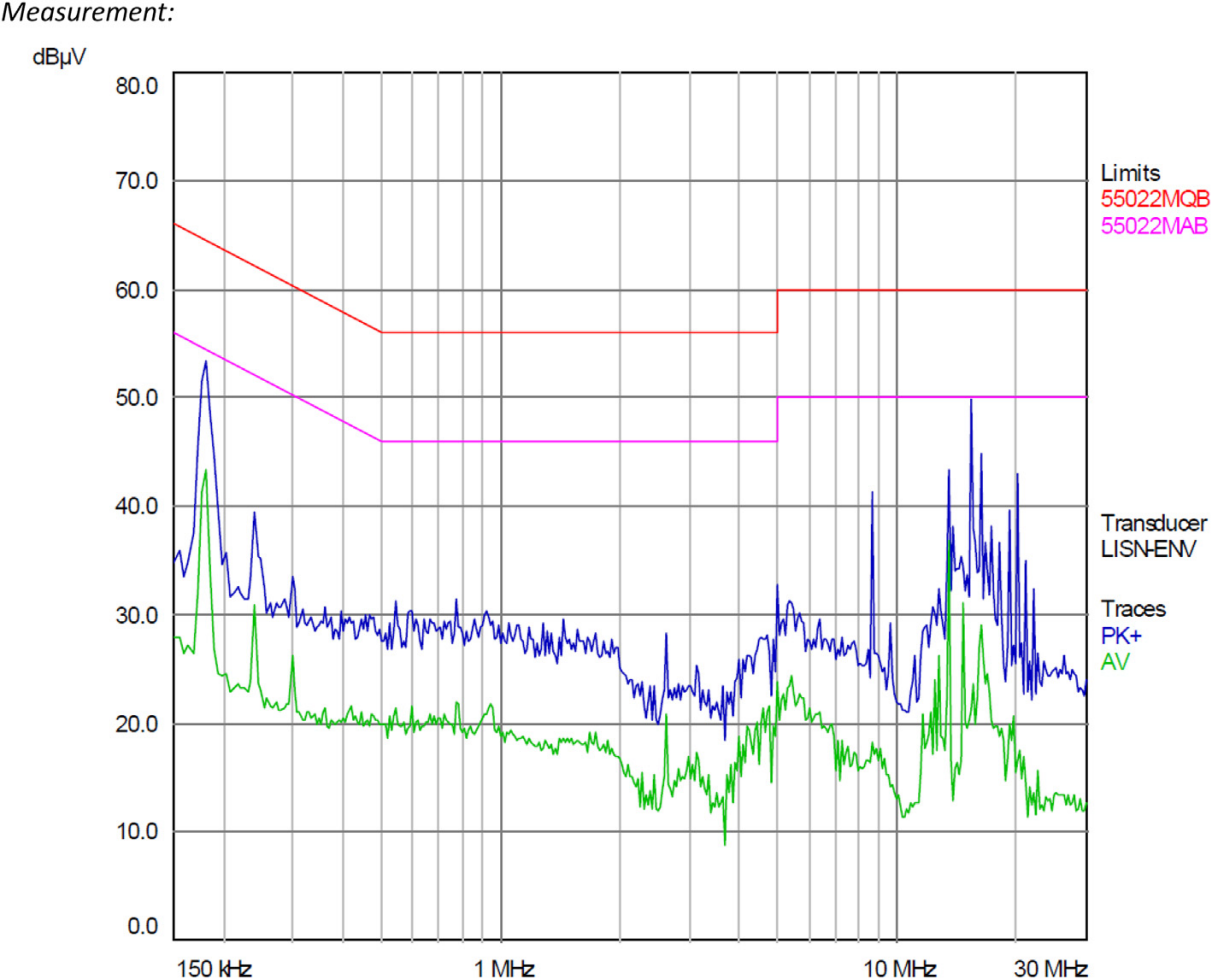
Conducted emission of electromagnetic noises from ATMO-Vent.

The conducted emission tests (Fig. 26) concluded that ATMO-Vent conductive emissions are well below the threshold set by the EN 55,022 Conducted Mains Class B standards.

#### 8.4.4 Radiated immunity

Test Specification: EN 61000–4 Radiated Immunity

Scan settings

80 MHz–2.7 GHz at 10 V/m 80% AM (requirements for Life Support). This scan setting was selected according to the guidelines provided by IEC61000-4-3 Radiated RF Immunity under IEC60601-1: Medical Design Standards for Power Supplies.

Measurement

**Fig. 27:**
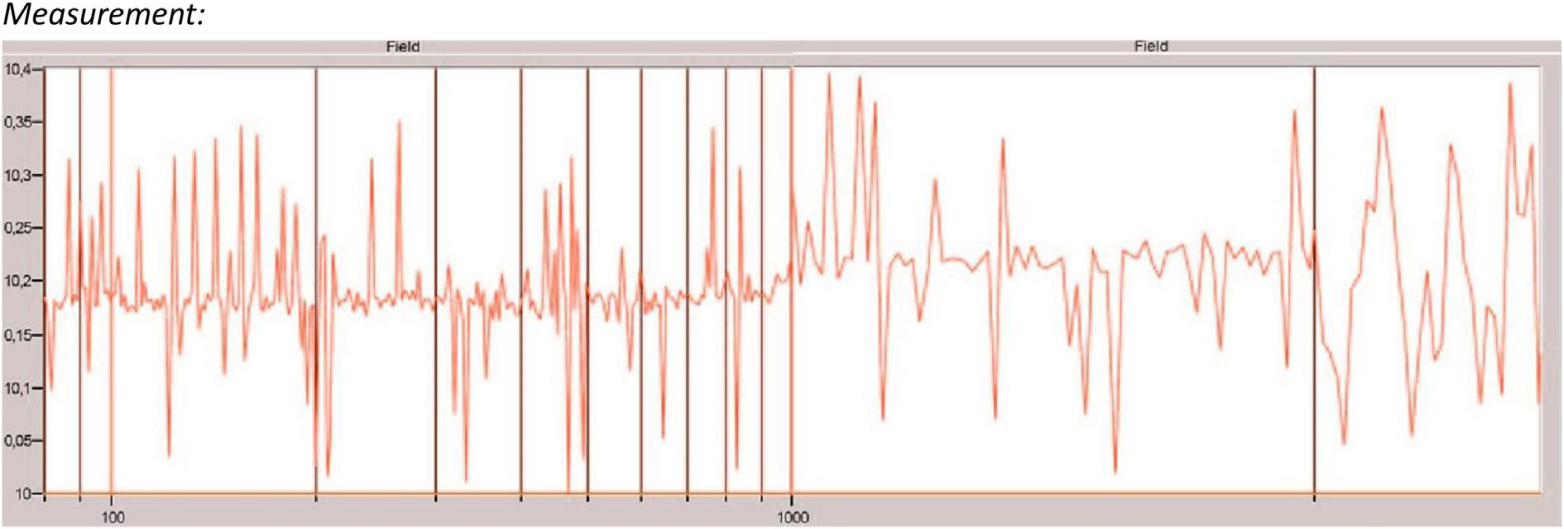
Radiated immunity test to verify the robustness of ATMO-Vent.

The radiated immunity tests (Fig. 27) concluded that ATMO-Vent operation was not affected on subjection to RF at 10 V/m in the frequency range from 80 MHz to 2.7 GHz.

## 9 Conclusion

ATMO-Vent has been carefully designed in consideration with the minimal clinical requirements of ventilators, using the UK-MHRA RMVS document as a guideline. The design also ensures that the materials and components used are readily available in the commercial market and could be assembled with minimal investment of time and resources. The design is modular, and components could be substituted with alternatives found in the local market specific to the installation of the ventilator. ATMO-Vent’s modes of operation have been tested using a mannequin and a simulated lung. The electronics integrity has been verified with EMC/EMI testing in an anechoic chamber in consideration with the IEC 60601 specifications. In the following version of ATMO-Vent, a higher stroke speed linear actuator will be used to achieve a higher tidal volume with a set frequency, and further tests with a lung simulator will be performed in order to quantify the output respiration parameters accurately.

The future line of work of action of ATMO-Vent are:

1. Robustness for long-sustained operation needs to be tested: when applied in clinical use, mechanical ventilators may be typically needed for a couple of weeks, and this requires about a million cycles of assisted ventilation. During this time the configuration of the ventilator needs to be adapted, for diagnosis and evaluation, and be accommodated to the evolving condition of the patient which may get worse or improve and eventually lead to retire the supporting ventilation. Thus, both the mechanical, electrical and software robustness needs to be tested on a long-time test.
2. Certify the equipment for its use in healthcare facilities. Although the design of this ventilator complies with the UK Medicines & Healthcare products Regulatory Agency (UK-MHRA) guidelines, the equipment needs to be certified according to the industrial and medical standards ISO 13485:2016(E). Although its design is in everything equal to existing commercial ventilators, before its operation in clinical use, it needs to be tested with trained healthcare personnel, to make sure that the information displayed in the screen, and the operating software interface is intuitive and similar to what is customarily used. The ventilator should also be subjected to Closed Suctioning Test to ensure continuous ventilator operation during the suctioning procedure. The closed suction is done to remove tracheal secretions through the endotracheal tube in mechanically ventilated patients.

ATMO-Vent can be used in the future as support for other respiratory diseases. Oxygen therapy coupled with mechanical ventilation is meant to support patients so that an adequate oxygen saturation (>88%) in arterial blood is maintained. The ability of ATMO-Vent to provide non-invasive assisted ventilation support with control over the fraction of inspired oxygen can help patients who are in the development stage of respiratory distress. This shall ensure that patients who develop ARDS could be attended with a full-fledged ventilator. Besides this, and beyond its clinical use, ATMO-Vent will be miniaturized and used for its future usage as 1) portable life-support equipment for long-inhabited environments without rapid access to hospitals (emergency clinics in rural environments, ships, camping sites, migrant- settlements, military or scientific bases in remote regions); 2) life support system for Space applications using space-qualified components.

## 10 Disclaimer

The designs and other information (the “Design”) made available in this article is at an early stage of development. Accordingly, specific results are not be guaranteed, and the Design provided here is provided “AS IS” and without any express or implied warranties, representations or undertakings. As examples, but without limiting the foregoing, the University of Aberdeen, Luleå University of Technology, Instituto Andaluz de Ciencias de la Tierra (CSIC-UGR), Centro de Astrobiología (CSIC-INTA) and their employees and students do not give any warranty or guarantee that the Design is of merchantable or satisfactory quality, is fit for any particular purpose, complies with any sample or description including the requirements for medical device registration, or are viable, uncontaminated, safe or non-toxic, accurate, up to date or complete. The University of Aberdeen, Luleå University of Technology and the authors have not performed any searches or investigations into the existence of any third-party rights that may affect the Design. Anyone may use the Design entirely at their own risk, and the University of Aberdeen, Luleå University of Technology, Instituto Andaluz de Ciencias de la Tierra (CSIC-UGR), Centro de Astrobiología (CSIC-INTA) and/or the authors are not liable for such use of the Design, including without limitation any direct or indirect losses. Any users of the design should appropriately attribute the author.

## Declaration of Competing Interest

The authors declare that they have no known competing financial interests or personal relationships that could have appeared to influence the work reported in this paper.
